# Supplementary material for: Endothelial Pim3 kinase protects the vascular barrier during lung metastasis
Source: Nat Commun. 2024 Dec 3;15:10514. doi: 10.1038/s41467-024-54445-1 (PMC11615401; doi:10.1038/s41467-024-54445-1)
Supplement: Supplementary file 3 — Description of Additional Supplementary Files [file 41467_2024_54445_MOESM3_ESM.pdf]

<u>Description of Additional Supplementary Files</u>

Supplementary Data 1. Cluster markers for integrated mouse lung ECs.
Top 50 cluster markers based on Adjusted P (AdjPval) value (two-sided Wilcoxon Rank-Sum Test) in mouse lung EC in the integrated scRNA-Seq datasets 1, 2 and 3 (three biological replicates) containing samples: B16-F10 6 h, 30 h and PBS (Ctrl).

Supplementary Data 2. Genes for enriched Cellchat communication pathways in the metastatic lungs (rCaps as signal sender).
Pathways (rCaps as signal sender) significantly upregulated in the metastatic lung EC 6 h after i.v, injection of melanoma cells to mice as compared to lung EC from the control-treated mice, ScRNA-Seq analysis of three independent datasets 1, 2 and 3.
Shown are adjusted P values (two-sided Wilcoxon Rank-Sum Test, significant P<0.05, non-significant ns).

Supplementary Data 3. DE genes between metastatic and control lung EC clusters.
Top 50 differentially expressed upregulated and downregulated genes between B16-F10 6h and PBS treated mouse lung EC. Adjusted P value (AdjPval) is based on two-sided Wilcoxon Rank-Sum Test.

Supplementary Data 4. Gene set enrichment analysis of KEGG pathways.
KEGG pathways identified based on DE genes derived from each cluster following comparison of the B16-F10 6h metastatic lung and control scRNASeq data, in genes min FC 0.1, min PCT 0.1 and Adj.Pval max 0.05.
Significant pathway selected based on FDR q-value max 25%, ordered based on NES.
Wilcoxon Rank-Sum Test was used to identify the differentially expressed (DE) genes. The gene set enrichment analysis (GSEA) utilised Kolmogorov-Smirnov test for enrichment score calculation and two-sided t-test for enrichment score significance testing. T-test results were FDR adjusted for multiple hypothesis comparisons.

Supplementary Data 5. Gene set enrichment analysis of Hallmark pathways.
Hallmarks were identified based on DE genes derived from each cluster following comparison of the B16-F10 6h metastatic lung and control scRNASeq data, in genes min FC 0.1, min PCT 0.1 and Adj.Pval max 0.05.
Significant pathway selected based on FDR q-value max 25%, ordered based on NES.
Wilcoxon Rank-Sum Test was used to identify the differentially expressed (DE) genes. The gene set enrichment analysis (GSEA) utilised Kolmogorov-Smirnov test for enrichment score calculation and two-sided t-test for enrichment score significance testing. T-test results were FDR adjusted for multiple hypothesis comparisons.

Supplementary Data 6. Python and R packages.
Packages containing the functions used in Python and R programming languages.Reference list is available at the supplementary information.pdf file.

Supplementary Movie 1: AZD-1208 induces gaps in CDH5 in mouse lungs.
PIM inhibitor AZD-1208 (30 mgkg-1, 100 µl per mouse) was orally administered daily for 5 days in C57BL/6 mice. CDH5 was stained in 150 µm thick lung sections. Z-stacks of confocal microscopic images were analyzed using Imaris. The images were 3D rendered and made into movies for better visualization using the CapCut application. CDH5 surface masking was used to visualise CDH5 positive junctions of alveolar capillaries. AZD-1208 induced gaps in the CDH5 lining (indicated by arrows).

Supplementary Movie 2: Continuous CDH5 staining in vehicle-treated mouse lungs.
Vehicle (100 µl per mouse) was orally administered daily for 5 days in C57BL/6 mice. CDH5 was stained in 150 µm thick lung sections. Z-stacks of confocal microscopic images were analyzed using Imaris. The images were 3D rendered and made into movies for better visualization using the CapCut application. CDH5 surface masking was used to visualize CDH5 positive junctions of alveolar capillaries.
