# Supplementary material for: Endothelial Pim3 kinase protects the vascular barrier during lung metastasis
Source: Nat Commun. 2024 Dec 3;15:10514. doi: 10.1038/s41467-024-54445-1 (PMC11615401; doi:10.1038/s41467-024-54445-1)
Supplement: Supplementary file 7 — Reporting Summary [file 41467_2024_54445_MOESM7_ESM.pdf]

nature portfolio

Corresponding author(s): Pipsa Saharinen

Last updated by author(s): 2024.10.31

Reporting Summary

Nature Portfolio wishes to improve the reproducibility of the work that we publish. This form provides structure for consistency and transparency in reporting. For further information on Nature Portfolio policies, see our Editorial Policies and the Editorial Policy Checklist.

Statistics

For all statistical analyses, confirm that the following items are present in the figure legend, table legend, main text, or Methods section.

n/a	Confirmed	
☐	☒	The exact sample size (*n*) for each experimental group/condition, given as a discrete number and unit of measurement
☐	☒	A statement on whether measurements were taken from distinct samples or whether the same sample was measured repeatedly
☐	☒	The statistical test(s) used AND whether they are one- or two-sided<br>*Only common tests should be described solely by name; describe more complex techniques in the Methods section.*
☒	☐	A description of all covariates tested
☐	☒	A description of any assumptions or corrections, such as tests of normality and adjustment for multiple comparisons
☐	☒	A full description of the statistical parameters including central tendency (e.g. means) or other basic estimates (e.g. regression coefficient) AND variation (e.g. standard deviation) or associated estimates of uncertainty (e.g. confidence intervals)
☐	☒	For null hypothesis testing, the test statistic (e.g. *F*, *t*, *r*) with confidence intervals, effect sizes, degrees of freedom and *P* value noted<br>*Give P values as exact values whenever suitable.*
☐	☒	For Bayesian analysis, information on the choice of priors and Markov chain Monte Carlo settings
☒	☐	For hierarchical and complex designs, identification of the appropriate level for tests and full reporting of outcomes
☒	☐	Estimates of effect sizes (e.g. Cohen's *d*, Pearson's *r*), indicating how they were calculated

Our web collection on statistics for biologists contains articles on many of the points above.

Software and code

Policy information about availability of computer code

Data collection	Mouse lung EC sorting, scRNASeq, CellRanger (10xGenomics, version 2.1.1), 3 independent experiments (experiment 1: Ctrl and 6h melanoma; experiment 2: Ctrl, 6h and 30h melanoma; experiment 3: ctrl and 6 h melanoma), on-line data from https://hlca.ds.czbiohub.org/ (Krasnow_2020 patients and all Banovich_Kropski_2020 patients, except patient VUHD689) and Lung tumor ECTax (mouse) (https://endotheliomics.shinyapps.io/lung_ectax/).
Data analysis	Seurat v5, SCtransform, RPCAintegration (satijalab.org/seurat/), CellChat (http://www.cellchat.org/), Chipster (Kallio, M.A. et al. Chipster: user-friendly analysis software for microarray and other high-throughput data. BMC Genomics 12, 507 (2011)), DESeq2 (v3.18), GSEA (v4.3.2) and other packages (https://bioconductor.org/, listed in Supplementary table 10).<br>Links to github for applicable codes: https://github.com/nmsantio/Mouse-melanoma-lung-EC-scRNASeq (murine i.v. melanoma lung data), https://github.com/nmsantio/Create-Seurat-Objects-from-on-line-sources (Lung EC Tax data convertion to Seurat Object), https://github.com/nmsantio/Integration-of-mouse-and-human-scRNASeq-data (human and mouse data integration), https://github.com/nmsantio/HLCA_scRNASeq_EC (sample processing from hlca). Permanent links https://doi.org/10.5281/zenodo.13734362; https://doi.org/10.5281/zenodo.13734391; https://doi.org/10.5281/zenodo.13734401; https://doi.org/10.5281/zenodo.13734414.<br>Analysis of EC monolayers using the ECIS software (v.1.2.252 O PC) and of microscopy data using Zen 3.1 (blue edition) software (Zeiss, Oberkochen, Germany), Pannoramic Viewer/ Case/ Slide Viewer (3D Histech), Imaris 10.0.0 Cell Imaging software (Oxford instruments), Fiji (1.48s, Fiji, Wayne Rashband, National Institutes of Health, Bethesda, MD, USA).<br>Statistical analysis using GraphPad Prism software (version 9.2.0, GraphPad Software, LLC, San Diego, CA, USA).

For manuscripts utilizing custom algorithms or software that are central to the research but not yet described in published literature, software must be made available to editors and reviewers. We strongly encourage code deposition in a community repository (e.g. GitHub). See the Nature Portfolio guidelines for submitting code & software for further information.

Data

Policy information about availability of data

All manuscripts must include a data availability statement. This statement should provide the following information, where applicable:

- Accession codes, unique identifiers, or web links for publicly available datasets
- A description of any restrictions on data availability
- For clinical datasets or third party data, please ensure that the statement adheres to our policy

The raw scRNASeq and bulk-RNA Seq data generated in this study are available in the GEO repository under accession codes GSE235394 [https://www.ncbi.nlm.nih.gov/geo/query/acc.cgi?acc=GSE235394] (scRNASeq dataset 1), GSE253749 [https://www.ncbi.nlm.nih.gov/geo/query/acc.cgi?acc=GSE253749] (scRNASeq dataset 2), GSE253751 [https://www.ncbi.nlm.nih.gov/geo/query/acc.cgi?acc=GSE253751] (scRNASeq dataset 3) and GSE25197 [https://www.ncbi.nlm.nih.gov/geo/query/acc.cgi?acc=GSE251979] (bulk mRNA-Seq). The numerical data generated in this study are provided in the Source Data file. The orthotopic Lewis Lung Carcinoma primary tumor EC scRNASeq data used in this study are available in the LungECTax database (https://endotheliomics.shinyapps.io/lung_ectax/) and human EC scRNASeq data used in this study are available in the Human Lung Cell Atlas database (https://hlca.ds.czbiohub.org/).

Research involving human participants, their data, or biological material

Policy information about studies with human participants or human data. See also policy information about sex, gender (identity/presentation), and sexual orientation and race, ethnicity and racism.

Reporting on sex and gender	-
Reporting on race, ethnicity, or other socially relevant groupings	-
Population characteristics	-
Recruitment	-
Ethics oversight	-

Note that full information on the approval of the study protocol must also be provided in the manuscript.

Field-specific reporting

Please select the one below that is the best fit for your research. If you are not sure, read the appropriate sections before making your selection.

☒ Life sciences ☐ Behavioural & social sciences ☐ Ecological, evolutionary & environmental sciences

For a reference copy of the document with all sections, see nature.com/documents/nr-reporting-summary-flat.pdf

Life sciences study design

All studies must disclose on these points even when the disclosure is negative.

Sample size	For in vitro experiments, a minimum of 3-4 independent experiments were performed to enable statistical testing.<br><br>For experiments involving mice, the estimation of group size was based on our previous experience in similar studies or pilot experiments. Therefore, depending on the experiment, a minimum of three mice per group was used, and the experiment was independently repeated. For some experiments, the group size was initially higher, typically 4 or 5 mice per group, which was repeated once.<br><br>For scRNASeq, three independent experiments were performed, pooling cells from 2-3 mice per sample.
Data exclusions	No relevant data was excluded
Replication	In vitro experiments for main figures were performed at least three times.
Randomization	Inbred mice were randomly allocated into experimental groups. Primary HUVEC and BEC cells on coverslips or culture plates were randomly assigned to experimental groups. To minimize lot-to-lot variability, experiments were independently repeated under consistent experimental conditions.
Blinding	Samples were named in numbers, imaged by authors K Ganesh and P Kaipainen, and analysed by N Santio.

Behavioural & social sciences study design

All studies must disclose on these points even when the disclosure is negative.

Study description	*Briefly describe the study type including whether data are quantitative, qualitative, or mixed-methods (e.g. qualitative cross-sectional, quantitative experimental, mixed-methods case study).*
Research sample	*State the research sample (e.g. Harvard university undergraduates, villagers in rural India) and provide relevant demographic information (e.g. age, sex) and indicate whether the sample is representative. Provide a rationale for the study sample chosen. For studies involving existing datasets, please describe the dataset and source.*
Sampling strategy	*Describe the sampling procedure (e.g. random, snowball, stratified, convenience). Describe the statistical methods that were used to predetermine sample size OR if no sample-size calculation was performed, describe how sample sizes were chosen and provide a rationale for why these sample sizes are sufficient. For qualitative data, please indicate whether data saturation was considered, and what criteria were used to decide that no further sampling was needed.*
Data collection	*Provide details about the data collection procedure, including the instruments or devices used to record the data (e.g. pen and paper, computer, eye tracker, video or audio equipment) whether anyone was present besides the participant(s) and the researcher, and whether the researcher was blind to experimental condition and/or the study hypothesis during data collection.*
Timing	*Indicate the start and stop dates of data collection. If there is a gap between collection periods, state the dates for each sample cohort.*
Data exclusions	*If no data were excluded from the analyses, state so OR if data were excluded, provide the exact number of exclusions and the rationale behind them, indicating whether exclusion criteria were pre-established.*
Non-participation	*State how many participants dropped out/declined participation and the reason(s) given OR provide response rate OR state that no participants dropped out/declined participation.*
Randomization	*If participants were not allocated into experimental groups, state so OR describe how participants were allocated to groups, and if allocation was not random, describe how covariates were controlled.*

Ecological, evolutionary & environmental sciences study design

All studies must disclose on these points even when the disclosure is negative.

Study description	*Briefly describe the study. For quantitative data include treatment factors and interactions, design structure (e.g. factorial, nested, hierarchical), nature and number of experimental units and replicates.*
Research sample	*Describe the research sample (e.g. a group of tagged Passer domesticus, all Stenocereus thurberi within Organ Pipe Cactus National Monument), and provide a rationale for the sample choice. When relevant, describe the organism taxa, source, sex, age range and any manipulations. State what population the sample is meant to represent when applicable. For studies involving existing datasets, describe the data and its source.*
Sampling strategy	*Note the sampling procedure. Describe the statistical methods that were used to predetermine sample size OR if no sample-size calculation was performed, describe how sample sizes were chosen and provide a rationale for why these sample sizes are sufficient.*
Data collection	*Describe the data collection procedure, including who recorded the data and how.*
Timing and spatial scale	*Indicate the start and stop dates of data collection, noting the frequency and periodicity of sampling and providing a rationale for these choices. If there is a gap between collection periods, state the dates for each sample cohort. Specify the spatial scale from which the data are taken*
Data exclusions	*If no data were excluded from the analyses, state so OR if data were excluded, describe the exclusions and the rationale behind them, indicating whether exclusion criteria were pre-established.*
Reproducibility	*Describe the measures taken to verify the reproducibility of experimental findings. For each experiment, note whether any attempts to repeat the experiment failed OR state that all attempts to repeat the experiment were successful.*
Randomization	*Describe how samples/organisms/participants were allocated into groups. If allocation was not random, describe how covariates were controlled. If this is not relevant to your study, explain why.*
Blinding	*Describe the extent of blinding used during data acquisition and analysis. If blinding was not possible, describe why OR explain why blinding was not relevant to your study.*

Did the study involve field work? ☐ Yes ☐ No

Field work, collection and transport

Field conditions	*Describe the study conditions for field work, providing relevant parameters (e.g. temperature, rainfall).*
Location	*State the location of the sampling or experiment, providing relevant parameters (e.g. latitude and longitude, elevation, water depth).*
Access & import/export	*Describe the efforts you have made to access habitats and to collect and import/export your samples in a responsible manner and in compliance with local, national and international laws, noting any permits that were obtained (give the name of the issuing authority, the date of issue, and any identifying information).*
Disturbance	*Describe any disturbance caused by the study and how it was minimized.*

Reporting for specific materials, systems and methods

We require information from authors about some types of materials, experimental systems and methods used in many studies. Here, indicate whether each material, system or method listed is relevant to your study. If you are not sure if a list item applies to your research, read the appropriate section before selecting a response.

Materials & experimental systems

n/a	Involved in the study	
☐	☒	Antibodies
☐	☒	Eukaryotic cell lines
☒	☐	Palaeontology and archaeology
☐	☒	Animals and other organisms
☒	☐	Clinical data
☒	☐	Dual use research of concern
☒	☐	Plants

Methods

n/a	Involved in the study	
☐	☒	ChIP-seq
☐	☒	Flow cytometry
☒	☐	MRI-based neuroimaging

Antibodies

Antibodies used	Antibodies are listed in Supplementary Tables 2 and 3.
Validation	All antibodies are validated in publications or citations referred to in the respective manufacturer's website in Supplementary Table 3.<br>A18 anti-a-catenin antibody was a kind gift from Dr. Nagafuchi (Nara Medical University) and is referred to by reference in the Supplementary Table 3.

Eukaryotic cell lines

Policy information about cell lines and Sex and Gender in Research

Cell line source(s)	The Mus musculus skin melanoma cell line B16-F10, mouse mammary gland carcinoma cell line 4T1 and Lewis Lung Carcinoma LL/2 (LLC1) (American Type Culture Collection, ATCC®CRL-6475, CRL-2539 and CRL-1642, Manassas, VA, USA), mouse B16-F10-eGFP-Puro (CL053, Imanis Life Sciences, Rochester, MN, USA), human umbilical vein ECs (HUVEC) (Cell Applications, Inc., San Diego, CA, USA) and HMVEC-dBlAd - Adult Human Dermal Microvascular ECs (a.k.a. blood microvascular ECs, BEC) (Lonza).
Authentication	Authenticated cell lines were ordered from ATCC and Imanis Life Sciences, and maintained in the research program, without further authentication. Primary endothelial cells were confirmed as endothelial cells using endothelial cell specific markers.
Mycoplasma contamination	no mycoplasma contamination
Commonly misidentified lines<br>(See ICLAC register)	-

Animals and other research organisms

Policy information about studies involving animals; ARRIVE guidelines recommended for reporting animal research, and Sex and Gender in Research

Laboratory animals	Male C57BL/6JRj mice and female BALB/cJRj mice were used between 8-12 weeks of age, both strains from Janvier labs (Le Genest-Saint-Isle, France). Mice were housed in individually ventilated cages with enrichment materials in a specific pathogen-free facility

	following the guidelines by the Federation of European Laboratory Animal Science Associations at 21+/-1 °C and relative humidity between 55+/-10 % % under 12 h dark/light cycle.
Wild animals	N/A
Reporting on sex	Male C57BL/6JRj mice were used. rCap marker expression and the effect of AZD-1208 on vascular leakage and metastasis were confirmed in female BALB/cJRj mice.
Field-collected samples	N/A
Ethics oversight	All experimental procedures involving mice were approved by the Project Authorization Board, Regional State Administrative Agency for Southern Finland and performed under the licence ESAVI/15852/2022.

Note that full information on the approval of the study protocol must also be provided in the manuscript.

Plants

Seed stocks	N/A
Novel plant genotypes	N/A
Authentication	N/A

ChIP-seq

Data deposition

☒ Confirm that both raw and final processed data have been deposited in a public database such as GEO.

☒ Confirm that you have deposited or provided access to graph files (e.g. BED files) for the called peaks.

Data access links
May remain private before publication.

The scRNASeq datasets are deposited in the GEO repository:
Experiment 1: GSE235394
Experiment 2:GSE253749
Experiment 3: GSE253751

The mRNASeq data are deposited in the GEO repository GSE251979.

Files in database submission

ScRNASeq

EXPERIMENT 1:
063 Ctrl
064 6h

Raw fasta files:
2019_063_S7_L001_I1_001.fastq.qz
2019_063_S7_L001_R1_001.fastq.qz
2019_063_S7_L001_R2_001.fastq.qz
2019_063_S7_L002_I1_001.fastq.qz
2019_063_S7_L002_R1_001.fastq.qz
2019_063_S7_L002_R2_001.fastq.qz
2019_064_S8_L001_I1_001.fastq.qz
2019_064_S8_L001_R1_001.fastq.qz
2019_064_S8_L001_R2_001.fastq.qz
2019_064_S8_L002_I1_001.fastq.qz
2019_064_S8_L002_R1_001.fastq.qz
2019_064_S8_L002_R2_001.fastq.qz

Processed data files:
063barcodes.tsv.gz
063features.tsv.gz
063matrix.mtx.gz
064barcodes.tsv.gz
064features.tsv.gz
064matrix.mtx.gz

EXPERIMENT 2:
052 Ctrl
051 6h
053 30h

Raw fasta files:
2023_051_S1_L001_I1_001.fastq.gz

2023_051_S1_L001_I2_001.fastq.gz
2023_051_S1_L001_R1_001.fastq.gz
2023_051_S1_L001_R2_001.fastq.gz
2023_051_S1_L002_I1_001.fastq.gz
2023_051_S1_L002_I2_001.fastq.gz
2023_051_S1_L002_R1_001.fastq.gz
2023_051_S1_L002_R2_001.fastq.gz
2023_051_S1_L003_I1_001.fastq.gz
2023_051_S1_L003_I2_001.fastq.gz
2023_051_S1_L003_R1_001.fastq.gz
2023_051_S1_L003_R2_001.fastq.gz
2023_051_S1_L004_I1_001.fastq.gz
2023_051_S1_L004_I2_001.fastq.gz
2023_051_S1_L004_R1_001.fastq.gz
2023_051_S1_L004_R2_001.fastq.gz
2023_052_S2_L001_I1_001.fastq.gz
2023_052_S2_L001_I2_001.fastq.gz
2023_052_S2_L001_R1_001.fastq.gz
2023_052_S2_L001_R2_001.fastq.gz
2023_052_S2_L002_I1_001.fastq.gz
2023_052_S2_L002_I2_001.fastq.gz
2023_052_S2_L002_R1_001.fastq.gz
2023_052_S2_L002_R2_001.fastq.gz
2023_052_S2_L003_I1_001.fastq.gz
2023_052_S2_L003_I2_001.fastq.gz
2023_052_S2_L003_R1_001.fastq.gz
2023_052_S2_L003_R2_001.fastq.gz
2023_052_S2_L004_I1_001.fastq.gz
2023_052_S2_L004_I2_001.fastq.gz
2023_052_S2_L004_R1_001.fastq.gz
2023_052_S2_L004_R2_001.fastq.gz
2023_053_S3_L001_I1_001.fastq.gz
2023_053_S3_L001_I2_001.fastq.gz
2023_053_S3_L001_R1_001.fastq.gz
2023_053_S3_L001_R2_001.fastq.gz
2023_053_S3_L002_I1_001.fastq.gz
2023_053_S3_L002_I2_001.fastq.gz
2023_053_S3_L002_R1_001.fastq.gz
2023_053_S3_L002_R2_001.fastq.gz
2023_053_S3_L003_I1_001.fastq.gz
2023_053_S3_L003_I2_001.fastq.gz
2023_053_S3_L003_R1_001.fastq.gz
2023_053_S3_L003_R2_001.fastq.gz
2023_053_S3_L004_I1_001.fastq.gz
2023_053_S3_L004_I2_001.fastq.gz
2023_053_S3_L004_R1_001.fastq.gz
2023_053_S3_L004_R2_001.fastq.gz

Processed data files:
051barcodes.tsv.gz
051matrix.mtx.gz
015features.tsv.gz
052barcodes.tsv.gz
052matrix.mtx.gz
052features.tsv.gz
053barcodes.tsv.gz
053matrix.mtx.gz
053features.tsv.gz

EXPERIMENT 3:
058 Ctrl
059 6h

2023_058_S4_L001_I1_001.fastq.gz
2023_058_S4_L001_I2_001.fastq.gz
2023_058_S4_L001_R1_001.fastq.gz
2023_058_S4_L001_R2_001.fastq.gz
2023_058_S4_L002_I1_001.fastq.gz
2023_058_S4_L002_I2_001.fastq.gz
2023_058_S4_L002_R1_001.fastq.gz
2023_058_S4_L002_R2_001.fastq.gz
2023_058_S4_L003_I1_001.fastq.gz
2023_058_S4_L003_I2_001.fastq.gz
2023_058_S4_L003_R1_001.fastq.gz
2023_058_S4_L003_R2_001.fastq.gz
2023_058_S4_L004_I1_001.fastq.gz

2023_058_S4_L004_I2_001.fastq.gz
2023_058_S4_L004_R1_001.fastq.gz
2023_058_S4_L004_R2_001.fastq.gz
2023_059_S5_L001_I1_001.fastq.gz
2023_059_S5_L001_I2_001.fastq.gz
2023_059_S5_L001_R1_001.fastq.gz
2023_059_S5_L001_R2_001.fastq.gz
2023_059_S5_L002_I1_001.fastq.gz
2023_059_S5_L002_I2_001.fastq.gz
2023_059_S5_L002_R1_001.fastq.gz
2023_059_S5_L002_R2_001.fastq.gz
2023_059_S5_L003_I1_001.fastq.gz
2023_059_S5_L003_I2_001.fastq.gz
2023_059_S5_L003_R1_001.fastq.gz
2023_059_S5_L003_R2_001.fastq.gz
2023_059_S5_L004_I1_001.fastq.gz
2023_059_S5_L004_I2_001.fastq.gz
2023_059_S5_L004_R1_001.fastq.gz
2023_059_S5_L004_R2_001.fastq.gz

Processed files:
058barcodes.tsv.gz
058matrix.mtx.gz
058features.tsv.gz
059barcodes.tsv.gz
059matrix.mtx.gz
059features.tsv.gz

Bulk Seq:

C1_EKRN230062195-1A_22FCJKLT3_L5_1.fq.gz
C2_EKRN230062196-1A_22FCJKLT3_L5_1.fq.gz
C3_EKRN230062197-1A_22FCJKLT3_L5_1.fq.gz
C4_EKRN230062198-1A_22FCJKLT3_L5_1.fq.gz
B1_EKRN230062199-1A_22FCJKLT3_L6_1.fq.gz
B2_EKRN230062200-1A_22FCJKLT3_L6_1.fq.gz
B3_EKRN230062201-1A_22FCJKLT3_L6_1.fq.gz
B4_EKRN230062202-1A_22FCJKLT3_L6_1.fq.gz
C1_EKRN230062195-1A_22FCJKLT3_L5_2.fq.gz
C2_EKRN230062196-1A_22FCJKLT3_L5_2.fq.gz
C3_EKRN230062197-1A_22FCJKLT3_L5_2.fq.gz
C4_EKRN230062198-1A_22FCJKLT3_L5_2.fq.gz
B1_EKRN230062199-1A_22FCJKLT3_L6_2.fq.gz
B2_EKRN230062200-1A_22FCJKLT3_L6_2.fq.gz
B3_EKRN230062201-1A_22FCJKLT3_L6_2.fq.gz
B4_EKRN230062202-1A_22FCJKLT3_L6_2.fq.gz

Genome browser session
(e.g. UCSC)

Provide a link to an anonymized genome browser session for "Initial submission" and "Revised version" documents only, to enable peer review. Write "no longer applicable" for "Final submission" documents.

Methodology

Replicates

Cells from two to three animals were combined per sample. Altogether three independent experiments.

Sequencing depth

10x sequencing

Antibodies

CD31-PE, BD Pharmingen, 553373 and CD45-APC, 561018 BD Biosciences, 1:300, FACS

Peak calling parameters

scRNAseq libraries were prepared using the Chromium Single Cell 3´ Reagent Kits v2 (10x Genomics; Pleasantom; CA, USA). The cell recovery aim for each library was 8000 cells. Libraries were sequenced in NovaSeq 6000 system (Illumina, San Diego, CA, USA) using S4 flow cell with read length of 28+8+89 followed by multiplexing and mapping to the mouse genome (build mm10) using CellRanger (10xGenomics, version 2.1.1.).

Data quality

Quality control of all scRNASeq datasets was performed by selecting genes expressed in more than 3 cells (min.cells = 3) and cells expressing 300 genes (min.features = 300) in which less than 20 % of unique molecular identifiers (UMIs) were derived from the mitochondrial genome. Each sample was individually clustered for analysis of doublets.
Multiplet Rate (%) estimation guidelines from 10xGenomics were followed when adjusting the expected doublet percentage (https://kb.10xgenomics.com/hc/en-us/articles/360001378811-What-is-the-maximum-number-of-cells-that-can-be-profiled). DoubletFinder was used to identify the doublets (pN = 0.25). The pK value was adjusted per sample.

Software

Sequencing was performed by the Sequencing laboratory of Institute for Molecular Medicine Finland FIMM Technology Centre, University of Helsinki. The CellRanger software (10xGenomics) was used for generation of expression matrices. Data were aggregated using Cell Ranger software, while processing of the raw data was continued in R versions ranging from 3.6.2. to 4.2.2 (www.r-

project.org) by utilizing Rstudio 2021.09.2+382 (rstudio.com) and the package Seurat (functions: Read10x, CreateSeuratObject) according to instructions by the Satija laboratory (https://satijalab.org/seurat/).

Flow Cytometry

Plots

Confirm that:

☒ The axis labels state the marker and fluorochrome used (e.g. CD4-FITC).

☒ The axis scales are clearly visible. Include numbers along axes only for bottom left plot of group (a 'group' is an analysis of identical markers).

☒ All plots are contour plots with outliers or pseudocolor plots.

☒ A numerical value for number of cells or percentage (with statistics) is provided.

Methodology

Sample preparation

For preparation of single cell suspensions, mouse tissues were minced on ice and digested for 1 h at 37°C using 1 mg ml-1 collagenase Type I (Gibco/Thermo Fisher Scientific), 0.1% BSA and 7.5 µl ml -1 of DNAseI (Roche, Basel, Switzerland) in DPBS (Gibco/Thermo Fisher Scientific). Thereafter, samples were vortexed and passed through a 40 or 70 µm Nylon cell strainer (Cornin Life Sciences or Thermo Fisher Scientific), centrifuged for 7 min, the cells were resuspended into red blood cell (RBC) lysis buffer (155 mM NH4Cl, 12 mM NaHCO3, 0.1 mM EDTA) for 2 min and washed using 0.1% BSA, 2 mm EDTA in PBS (FACS buffer). For liver samples, the RBC lysis was performed twice. Cells were stained using anti-CD31-PE and CD45-APC-conjugated antibody in FACS buffer for 30 min to label ECs, washed twice with FACS buffer and sorted using BD InfluxTM (BD Biosciences, Franklin Lakes, NJ, USA), PE+, APC- cells were collected along with prelabelled CellTrackerGreen+ melanoma cells (CellTracker Green CMFDA Dye C2925, Thermo Fisher Scientific) prior to injection to mice.

Instrument

BD InfluxTM (BD Biosciences, Franklin Lakes, NJ, USA), cytometer serial # X646500S7001

Software

BD FACSTM Software 1.2.0.142

Cell population abundance

6-12% PE positive from all cells

Gating strategy

Gatings are explained with example images in Supplementary figure 2b.

☒ Tick this box to confirm that a figure exemplifying the gating strategy is provided in the Supplementary Information.
