# Supplementary material for: Endothelial Pim3 kinase protects the vascular barrier during lung metastasis
Source: Nat Commun. 2024 Dec 3;15:10514. doi: 10.1038/s41467-024-54445-1 (PMC11615401; doi:10.1038/s41467-024-54445-1)
Supplement: Supplementary file 1 — Supplementary Information [file 41467_2024_54445_MOESM1_ESM.pdf]

Supplementary Information for

Endothelial Pim3 kinase protects the vascular barrier during lung metastasis

Niina M. Santio[#], Keerthana Ganesh[#], Pihla P. Kaipainen, Aleksi Halme, Fatemeh Seyednasrollah, Emad Arbash, Satu Hänninen, Riikka Kivelä, Olli Carpen & Pipsa Saharinen*

Supplementary Figures 1-13

Supplementary Tables 1-4

Supplementary Note 1

Supplementary references

Santio et al. Endothelial Pim3 kinase protects the vascular barrier during lung metastasis.
Supplementary Information; Supplementary Figures 1-13

Supplementary Figures 1-13

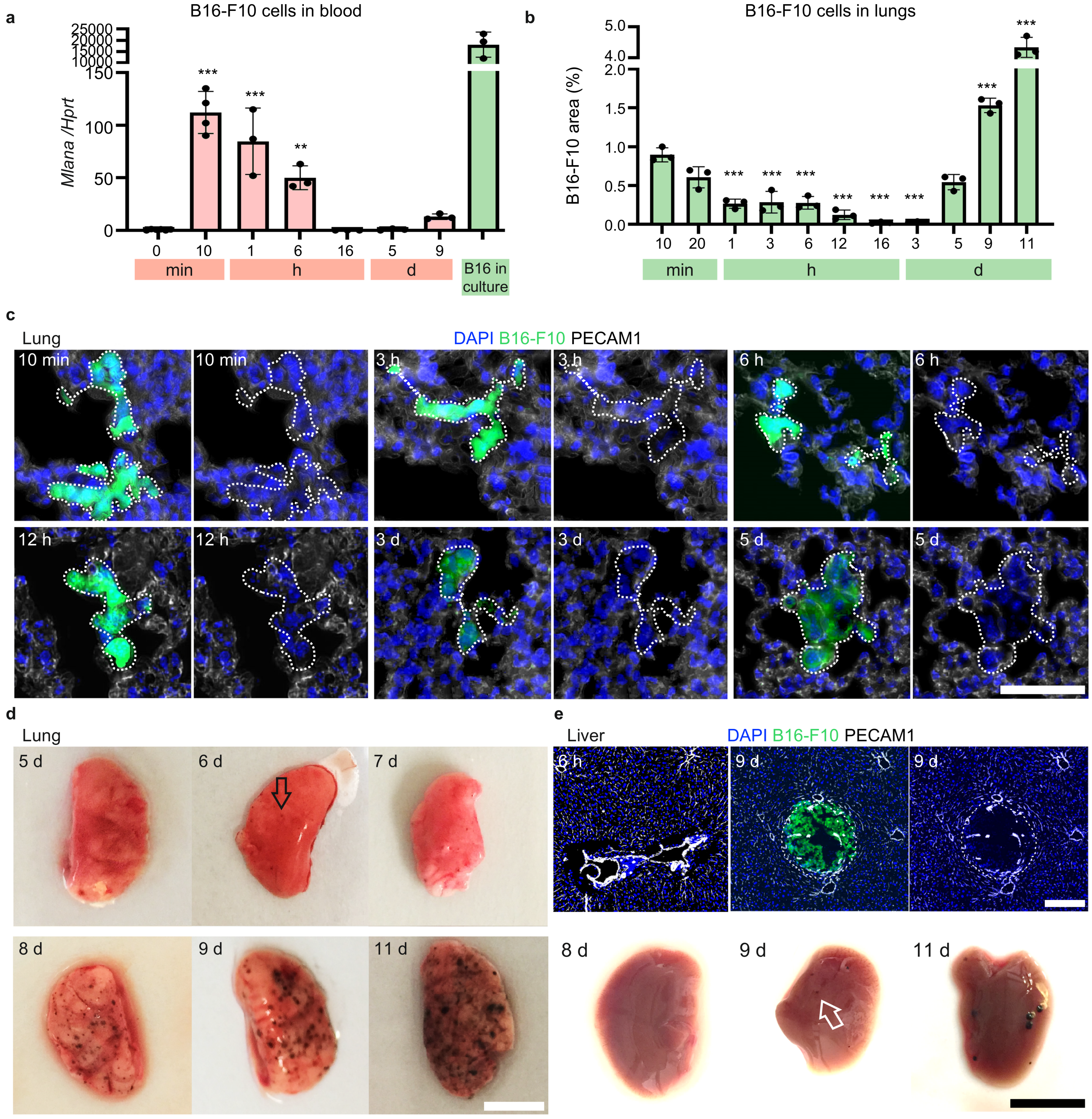

Supplementary Figure 1. Time-course of B16-F10 metastatic colonization in C57BL/6 mice. CellTracker Green labelled metastatic B16-F10 melanoma cells (200,000) were intravenously (i.v.) injected into C57BL/6 mice and analysed at various time points. **a**. RT-qPCR of melanoma marker *Mlana* normalized to *Hprt* in whole blood samples at indicated time points after B16-F10 injection relative to control (0 min). Equal amount of RNA from cultured B16-F10 cells was used as a positive control. In each data point n = 3 mice per group. **b**. B16-F10 cells were quantified from lung sections based on fluorescent signal from CellTracker Green or PMEL antibody staining. ECs were stained using PECAM1 antibody. Shown is melanoma cell area per lung lobe area. n = 3 mice per group. **c**. Representative images from b at indicated time points. **d**. Photographs of lungs after B16-F10 i.v. injection at indicated time points. Melanoma nodules were visible from day 6 onwards (indicated by an arrow). **e**. PMEL immunohistochemistry of liver sections and photographs of livers at indicated times after B16-F10 i.v. injection in mice. Note that melanoma cells were detected at the liver surface from day 9 onwards (indicated by an arrow). Scale bars 50 μm (c), 5 mm (d), 200 μm (e, top), 10 mm (e, lower panel). One-way Anova with multiple comparison $p < 0.01$** and < 0.001***, significant differences shown in comparison to time point 0 (a) and 10 min (b). Data are presented as mean values +/- SD. Source data are provided as a Source Data file. *This is supportive data for Figure 1.*

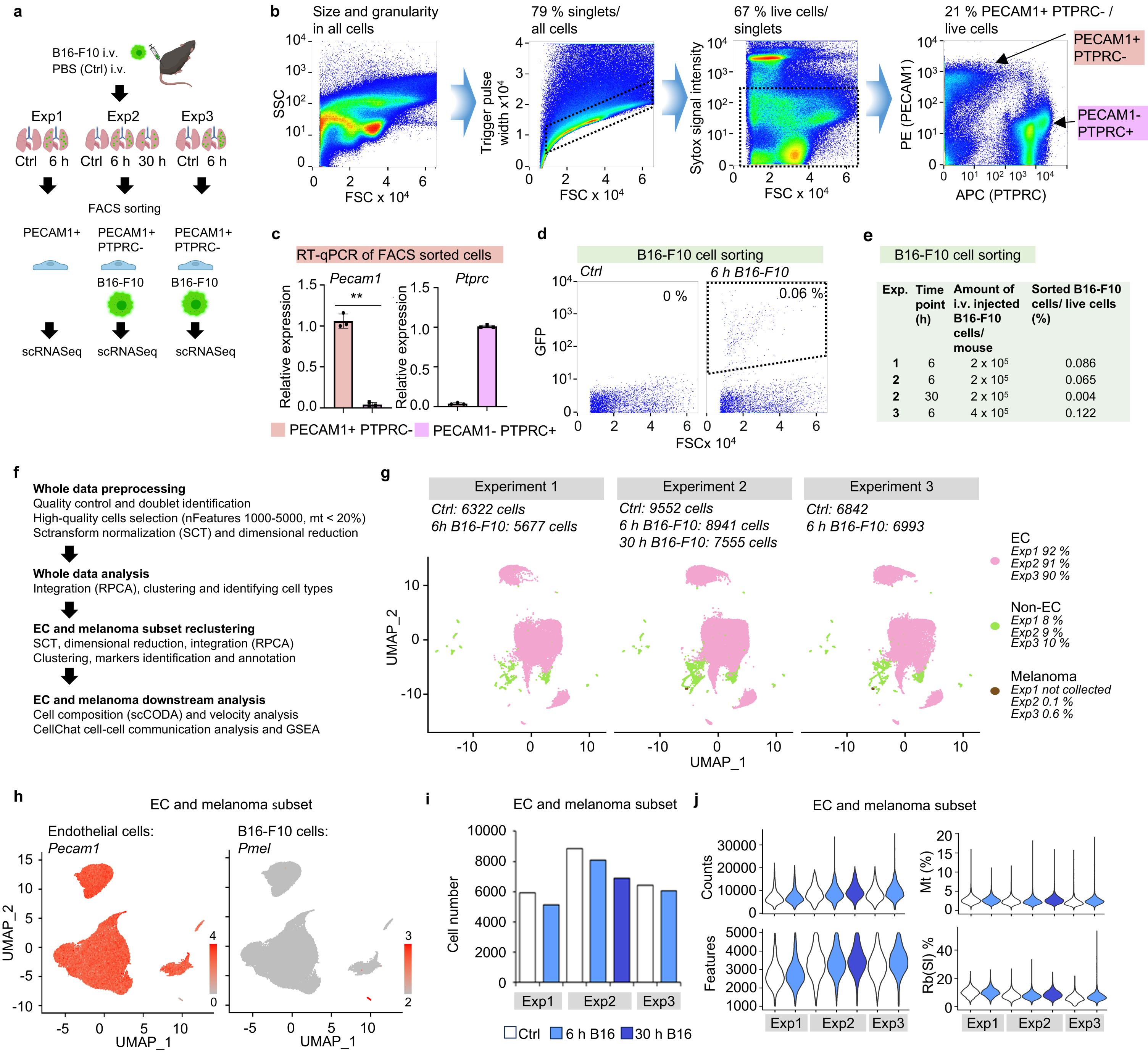

Supplementary Figure 2. scRNASeq sample preparation, quality controls and data analysis workflow. a. Sample preparation workflow. CellTracker Green labelled B16-F10 melanoma cells (200,000 in exp1-2, 400,000 in exp3) or PBS (ctrl) were intravenously (i.v.) injected into C57BL/6 mice. After 6 h and 30 h single cell suspensions were prepared from the lungs, stained using CD31-PE (a.k.a. PECAM1) and CD45-APC (a.k.a. PTPRC) antibodies and sorted using flow cytometry. Three independent experiments (n=3, Exp1-3) were performed, consisting of seven samples, each sample pooled from 2-3 mice. **b.** Representative image of cell sorting (exp 2, ctrl). Shown are granularity versus size (side scatter, SSC / forward scatter, FSC), along with singlet (Trigger pulse width) and viable cell detection using SYTOX™ Blue (ex 405 nm, em 435-485 nm) to detect dead cells. Endothelial cells (EC) were sorted as PECAM1+ (PE ex 561, em 570-600 nm) and PTPRC**-** (APC ex 640, em 655-685). **c.** RT-qPCR of EC and hematopoietic cell markers in the sorted PECAM1+ PTPRC- and PECAM1- PTPRC+ populations, normalized to *Hprt*. n=3 independent experiments. **d.** Melanoma cells were sorted based on CellTracker Green (ex 488, em 510-550 nm). Shown are examples from exp 2. **e.** Sorted B16-F10 cell content for each metastatic sample. **f.** scRNA-Seq data analysis workflow with Seurat 5 toolkit as the core framework. **g.** Uniform Manifold Approximation and Projection (UMAP) of all cells, split by experiment. **h.** Feature plots for EC and melanoma markers of the integrated EC and melanoma subsets from all samples. **i-j.** Cell number of the EC and melanoma cell subsets per sample (i) and quality metrics per sample (j). Data are presented as mean values +/- SD. Two-tailed unpaired t-test, $p < 0.01$**(c). Illustration created in Biorender.com (a). Source data are provided as a Source Data file. *This is supportive data for Figure 1.*

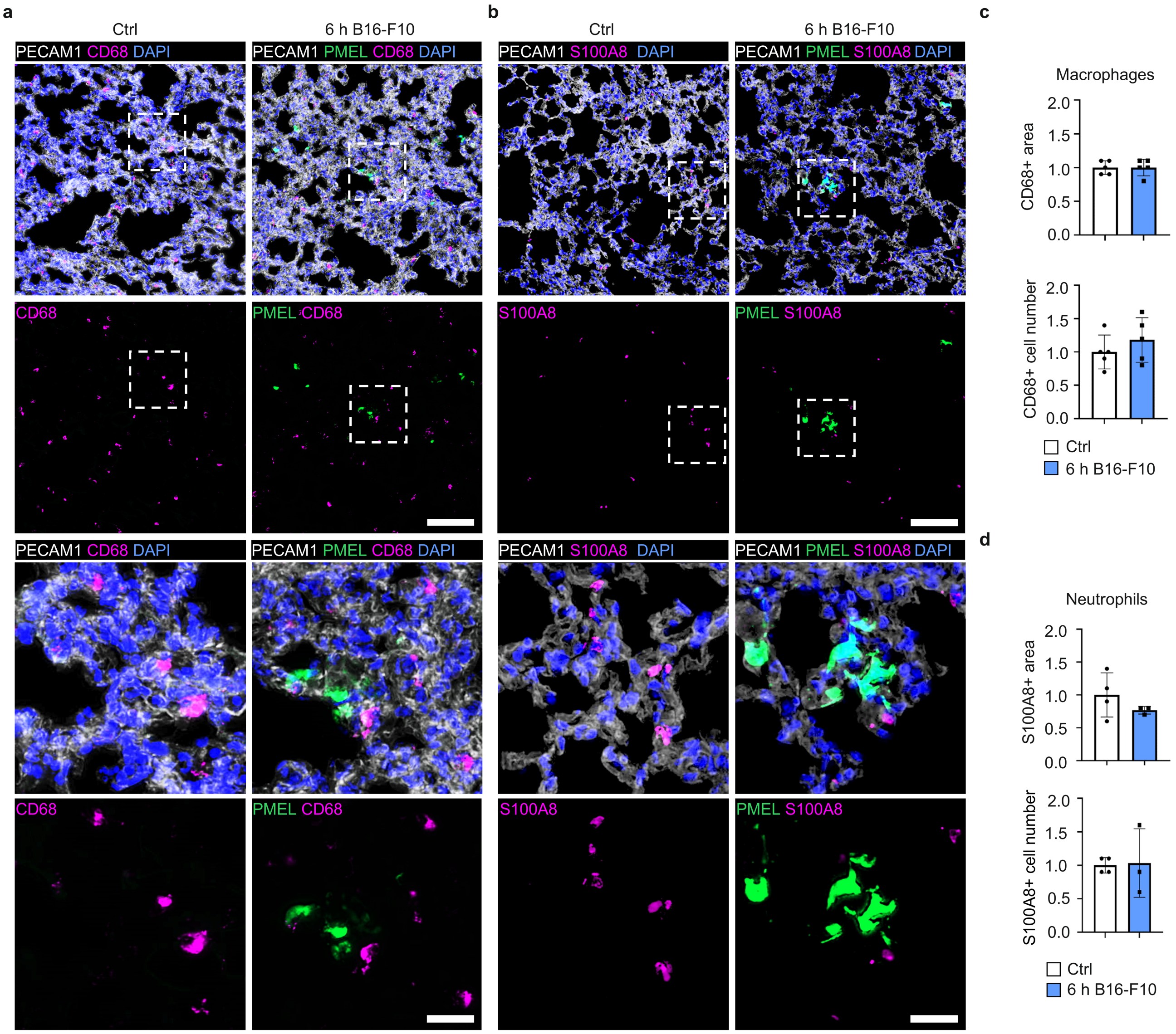

Supplementary Figure 3. No change in macrophage or neutrophil content in the lungs 6 h after i.v. injection of B16-F10 melanoma. B16-F10 melanoma cells (200,000) or PBS (Ctrl) were intravenously (i.v.) injected to C57BL/6 mice for 6 h. **a-b.** Macrophages were stained using CD68 (a) and neutrophils using S100A8 (b) in lung sections from control and B16-F10 injected mice. Endothelial cells were stained using PECAM1 and melanoma cells using PMEL antibodies. Representative images are shown. **c-d**. Quantification of macrophages (n=5 mice per group) (c) and neutrophils (n=4 Ctrl mice, n=3 B16-F10 injected mice) (d) per lung lobe area normalized to ctrl. Data are presented as mean values +/- SD. No significant differences. Scale bars 100 μm (a, b), 25 μm in close-up images (a, b). Source data are provided as a Source Data file. *This is supportive data for Figures 1-2.*

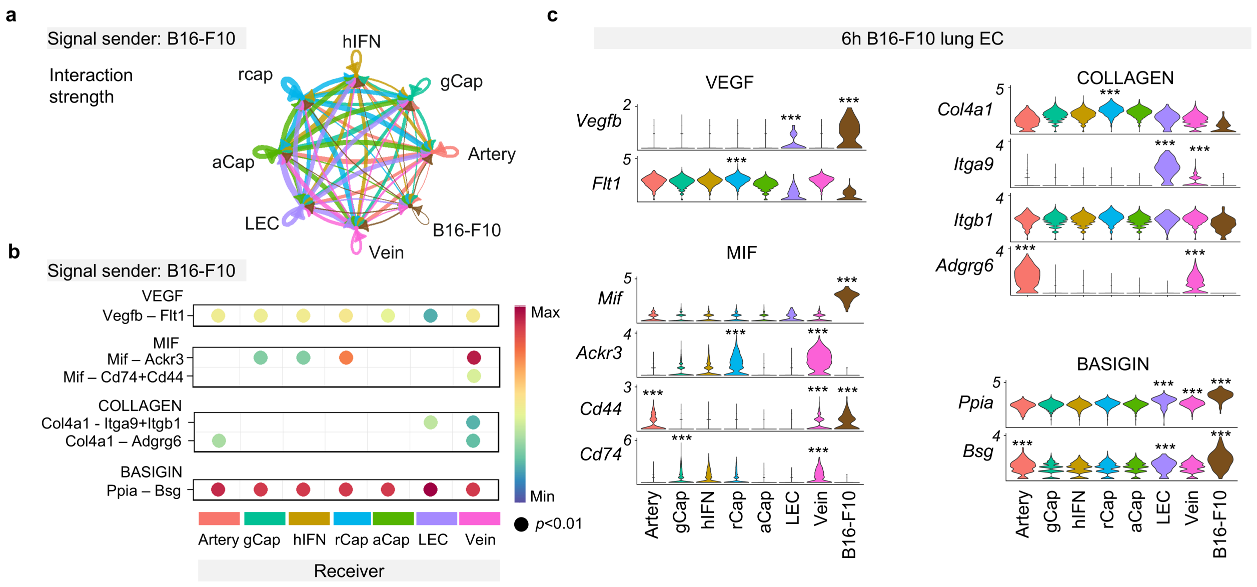

Supplementary Figure 4. Melanoma and EC–mediated cell communication pathways. CellChat analysis of cell-cell communication from B16-F10 to endothelial cell (EC) clusters 6 h after intravenous (i.v.) B16-F10 injection in mice (cells pooled from 3 mice). **a.** Circle plot of interactions between cell clusters. **b.** Bubble plot of predicted upregulated ligand-receptor interactions between B16-F10 and ECs, 6 h after B16-F10 injection, p value < 0.01. **c.** Violin plots of CellChat pathways. Aerocyte capillary, aCap; general capillary, gCap; high interferon EC, hIFN; lymphatic EC, LEC; reactive capillary, rCap. Wilcoxon rank-sum test, $p < 0.01$*** (c). Source data are provided as a Source Data file. *This is supportive data for Figure 2.*

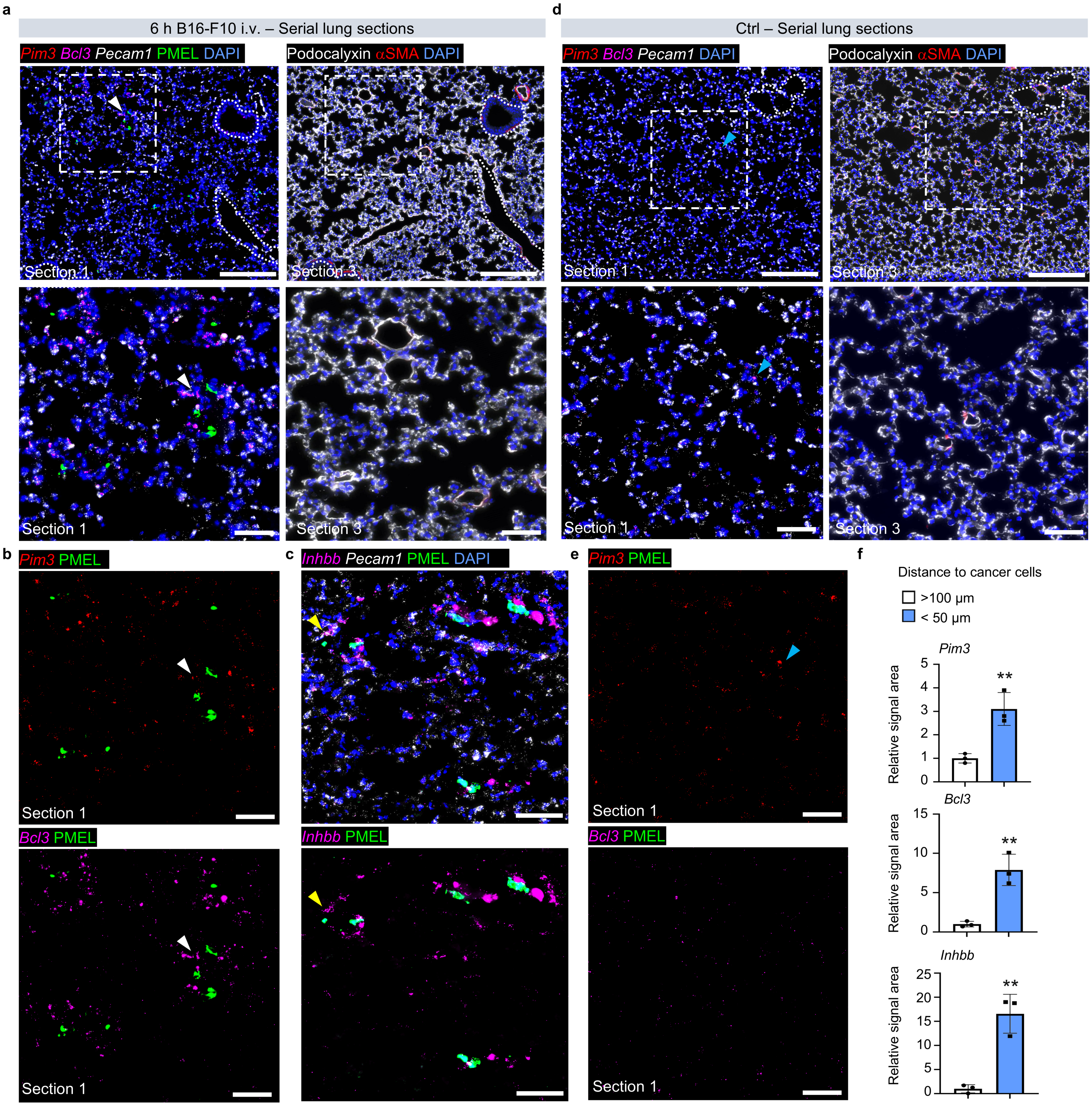

Supplementary Figure 5. rCap marker genes are enriched in the vicinity of cancer cells in the metastatic lungs. a-e. Paraffin sections were prepared from mouse lungs 6 h after intravenous (i.v.) injection of 200,000 B16-F10 melanoma cells (a, b, c) or PBS (Ctrl) (d-e). RNAscope^{TM} *in situ* hybridization (ISH) using probes for *Pim3-C1, Bcl3-C2* and *Pecam1-C3* (a, b, d, e) and for *Pim3-C1, Inhbb-C2 and Pecam1-C3* (c), was combined with anti-PMEL antibody staining of B16-F10 cells and nuclear staining by DAPI, as indicated. Additional serial sections were stained for Podocalyxin, to visualize ECs, and alpha-smooth muscle actin (αSMA) (serial section 3 in a, d). Shown are representative areas imaged using 3D Histech slide scanner. Areas indicated by rectangles in a and d (top row) are shown in higher magnification in a and d (lower row) and in b and e, respectively. **f**. *Pim3*, *Bcl3* and *Inhbb* signals were quantified from 7 areas (~100 µm x 100 µm) close to B16-F10 cells (distance < 50 µm from cancer cells) and compared to 7 areas distant to B16-F10 cells (distance > 100 µm from cancer cells) in mouse lungs 6 hours after B16-F10 i.v. injection and normalized to nuclear signal. n=3 mice per group. Two-tailed unpaired T-test $p < 0.01$**. Data are presented as mean values +/- SD. Scale bars 200 µm (top row in a, d), 50 µm (b, c, e, close-up images in a and d). White (a, b), yellow (c) and blue (d, e) arrowheads highlight ISH signal. IHC, immunohistochemistry. Source data are provided as a Source Data file. *This is supportive data for Figure 3.*

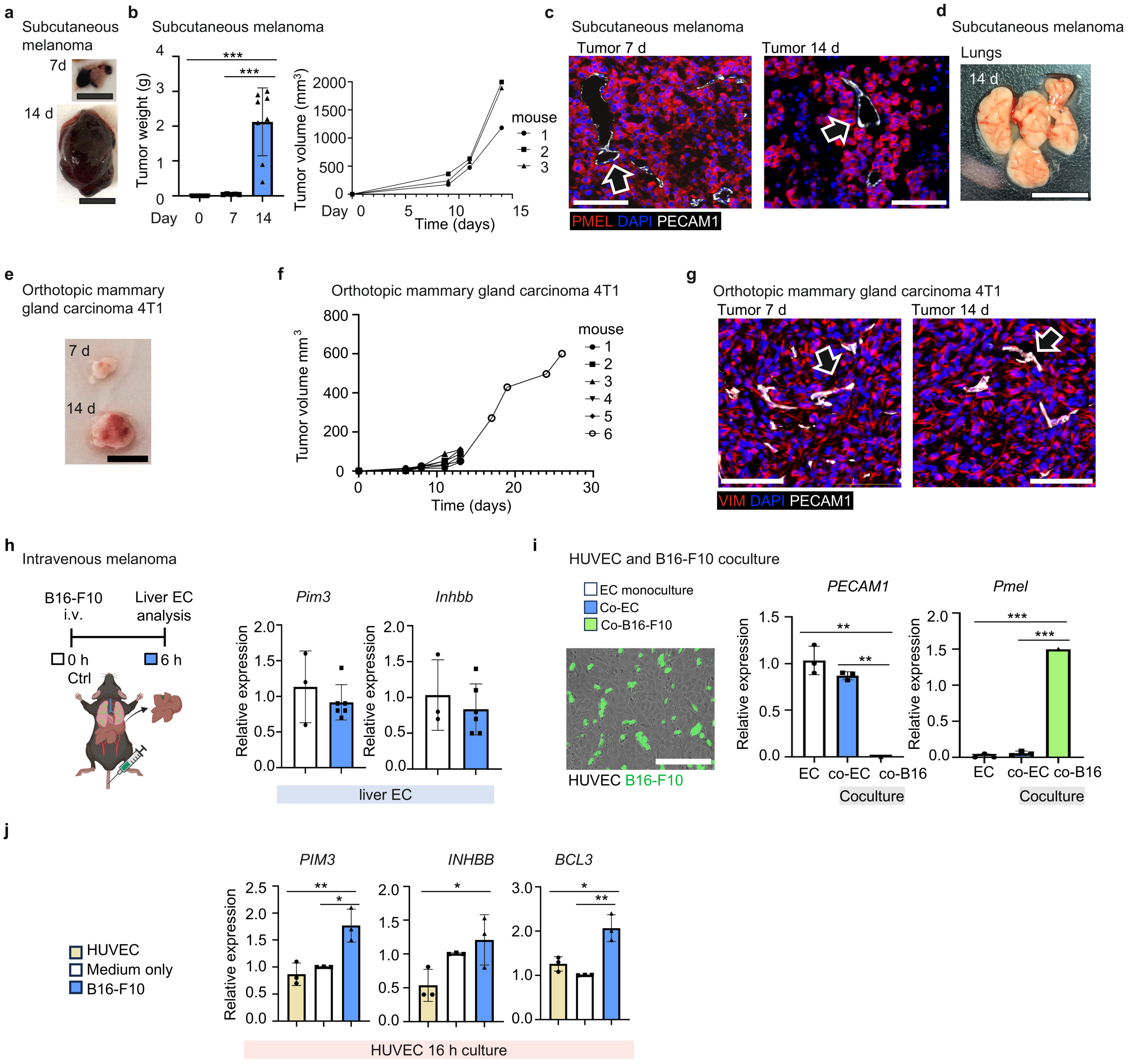

Supplementary Figure 6. Growth of subcutaneous B16-F10 melanoma and orthotopic mammary 4T1 and expression of rCap markers. a. Representative images of subcutaneous B16-F10 melanoma tumors at indicated times after tumor cell implantation. **b.** Tumor weight at day 7 and 14. n=8 mice, for three mice tumor volume by caliper was measured using formula: (width)2 x length/2**. c**. Staining of tumor vasculature for PECAM1, B16-F10 cells for PMEL, and nuclei using DAPI at indicated times. **d**. Photographs of lungs at day 14 (no metastases). **e.** Representative images of 4T1 orthotopic mammary tumors at indicated times after 4T1 implantation. **f.** Tumor volumes by caliper measurement. **g.** Staining of tumor vasculature for PECAM1, 4T1 cells for vimentin (VIM) and nuclei using DAPI. **h.** B16-F10 (n=6 mice) or PBS (control) (n=3 mice) were injected i.v. into C57BL/6 mice. RT-qPCR of isolated liver ECs for *Pim3* and *Inhbb* 6 h post injection, normalized to *Hprt*. **i.** Representative image of a coculture of human umbilical vein endothelial cells (HUVEC) and B16-F10 melanoma stably expressing GFP. Sorting of pure cell populations from cocultures (co-EC, co-B16) was confirmed by RT-qPCR for *PECAM1* and melanoma marker *PMEL,* normalized to *HPRT.* HUVEC monocultures were used as a control (EC, n=3 independent experiments). **j.** RT-qPCR of HUVECs treated for 16 h with conditioned medium derived from 48 h control cultures (HUVEC culture or medium only) or B16-F10 cell cultures, normalized to *HPRT*. n=3 independent experiments. One-way Anova with multiple comparison $p < 0.05$*, <0.01**, <0.001*** (i, j). Data are presented as mean values +/- SD. Scale bars 1 cm (a, d, e), 100 μm (c, g), 200 μm (i). Arrows indicate tumor vessels. Illustration created in Biorender.com (h). Source data are provided as a Source Data file. *This is supportive data for Figure 4.*

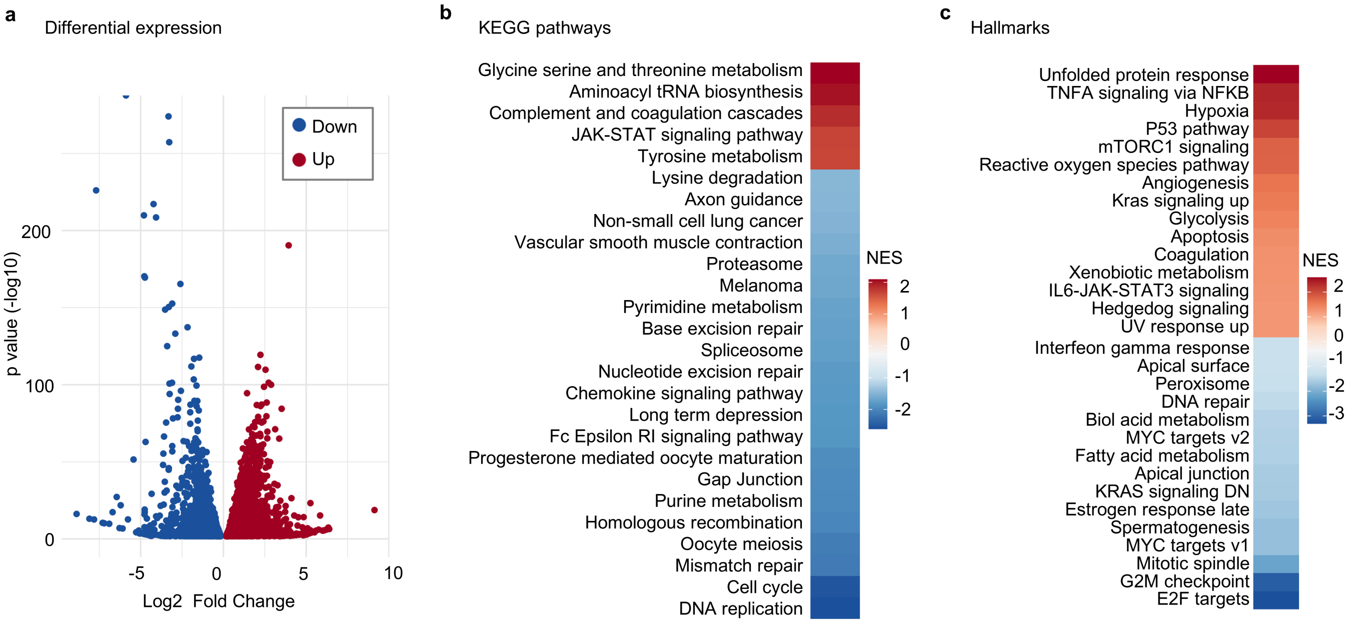

Supplementary Figure 7. Gene expression analysis of HUVECs in response to melanoma secretome using bulk mRNA-Seq. HUVECs were incubated for 16 hours in conditioned medium cultured for 48 h with B16-F10 melanoma cells or empty plate, as a control. Total RNA from HUVECs was subjected to bulk-RNA seq (n=4 independent experiments). **a.** Volcano plot visualization of the significant differentially expressed (DE) genes in B16-F10 conditioned medium-treated HUVECs as compared to control (adjusted *p*-value < 0.05). **b-c.** Gene set enrichment analysis of all significantly enriched Kyoto Encyclopedia of Genes and Genomes (KEGG) pathways (b) and Hallmarks pathways of the DE genes (FDR < 25%). (c). *This is supportive data for Figure 4.*

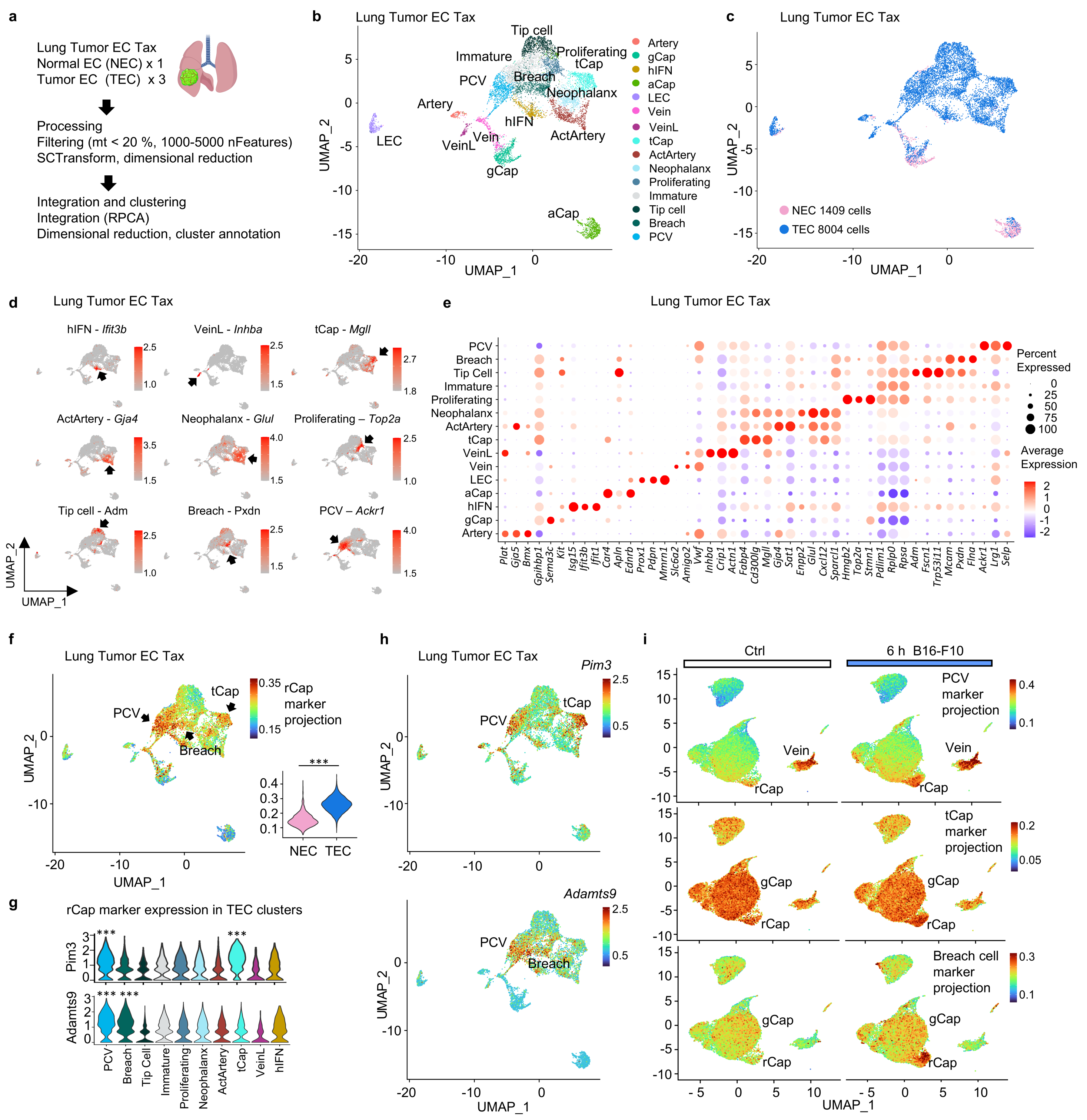

Supplementary Figure 8. rCap markers are enriched in the lung tumor vasculature. a. For analysis of rCap marker expression in mouse tumor endothelial cells (ECs), we utilized the raw data from Lung Tumor EC Tax. After filtering and normalization, the final dataset contained small number of normal lung ECs (NEC) and higher number of tumor ECs (TEC) derived from 3 mice orthotopically injected with Lewis Lung Carcinoma cells. **b-c.** Clusters were annotated based on Goveia et al. [1]. Shown are integrated data visualization after Uniform Manifold Approximation and Projection (UMAP). **d-e.** Main markers are shown for selected clusters using feature plots and for all clusters using dotplot. **f-g**. Top 50 rCap markers from our mouse data were projected to the data derived from Lung EC Tax (NEC and TEC) (f). Ucell scores showing highest rCap marker expression in the post-capillary venules (PCV), breach ECs and tumor capillary ECs (tCap) (g). **h.** Feature plot visualization of rCap markers *Pim3* and *Adamts9* in the LungECTax data. **i.** PCV, tCap and breach cell markers based on Goveia et al. [1] were visualized in EC subset of the control lungs and lungs i.v. injected with B16-F10 at 6 h time point. Wilcoxon rank-sum test, $p < 0.001$***. ActArtery, activated artery; aCap, aerocyte capillary; hIFN, high interferon; gCap, general capillary; PCV, post-capillary vein; tCap, tumor capillary; VeinL, large vein. Illustration created in Biorender.com (a). *This is supportive data for Figures 1-4.*

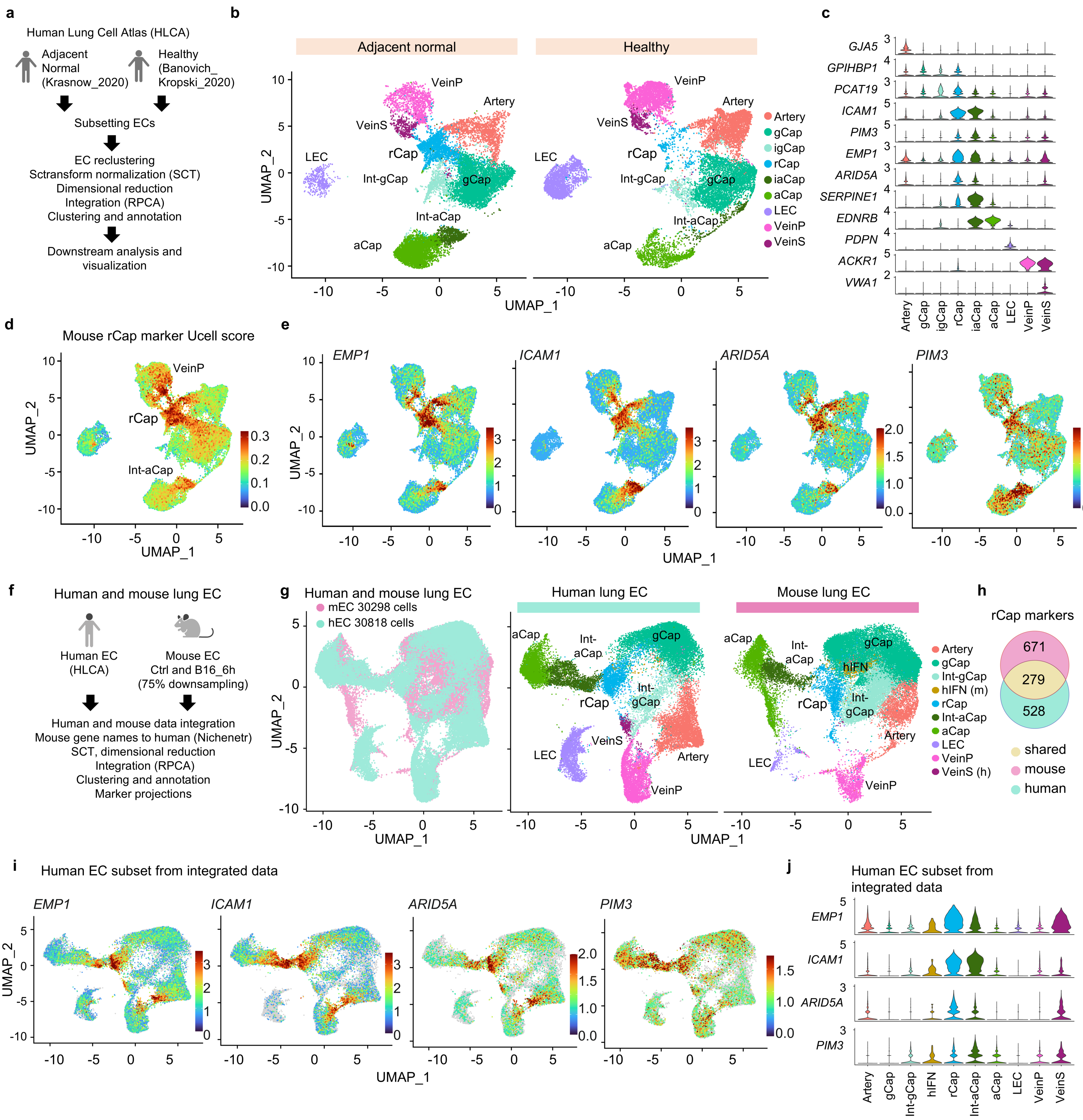

Supplementary Figure 9. Cancer reactive endothelial cells are found in human lungs. a. 10x Genomics scRNA-Seq data from human lungs was downloaded from Human Lung Cell Atlas (HLCA) through https://hlca.ds.czbiohub.org/ website. EC subsets from healthy lungs (Banovich_Kropski, n = 36) and non-carcinoma lung tissue adjacent to a lung tumor from carcinoma patient (Travaglini, n = 3) were selected based on previous annotations for downstream analysis workflow. **b.** Uniform Manifold Approximation and Projection (UMAP) of the human EC subset data. **c**. Previously identified human EC markers and novel markers for cancer reactive capillary ECs (rCap). **d.** Projection of top 50 mouse rCap markers to the human EC data. **e**. Feature plot visualization of the selected human rCap markers. **f**. Integration of the human lung EC data with our mouse lung EC data originating from the intravenously (i.v.) injected datasets (6 h B16-F10 and Ctrl, n = 3 independent experiments, 75% downsampling). **g.** UMAP visualization of the human and mouse EC data (human 30818 and mouse 30298 cells). **h.** Venn diagram of the mouse and human rCap markers from the integrated data (Log2 FC > 0.1, min.pct 0.1, Adj *p*-value < 0.05, Wilcoxon rank-sum test). **i-j.** Feature plot (i) and violin plot (j) visualization of the selected human rCap markers in the human EC subset after data integration. Aerocyte capillary, aCap; general capillary, gCap; high interferon EC, hIFN; intermediate, int; lymphatic EC, LEC; pulmonary vein, VeinP; reactive capillary EC, rCap; systemic vein, VeinS. Illustrations created in Biorender.com (a, f). Source data are provided as a Source Data file. *This is supportive data for Figures 1-4.*

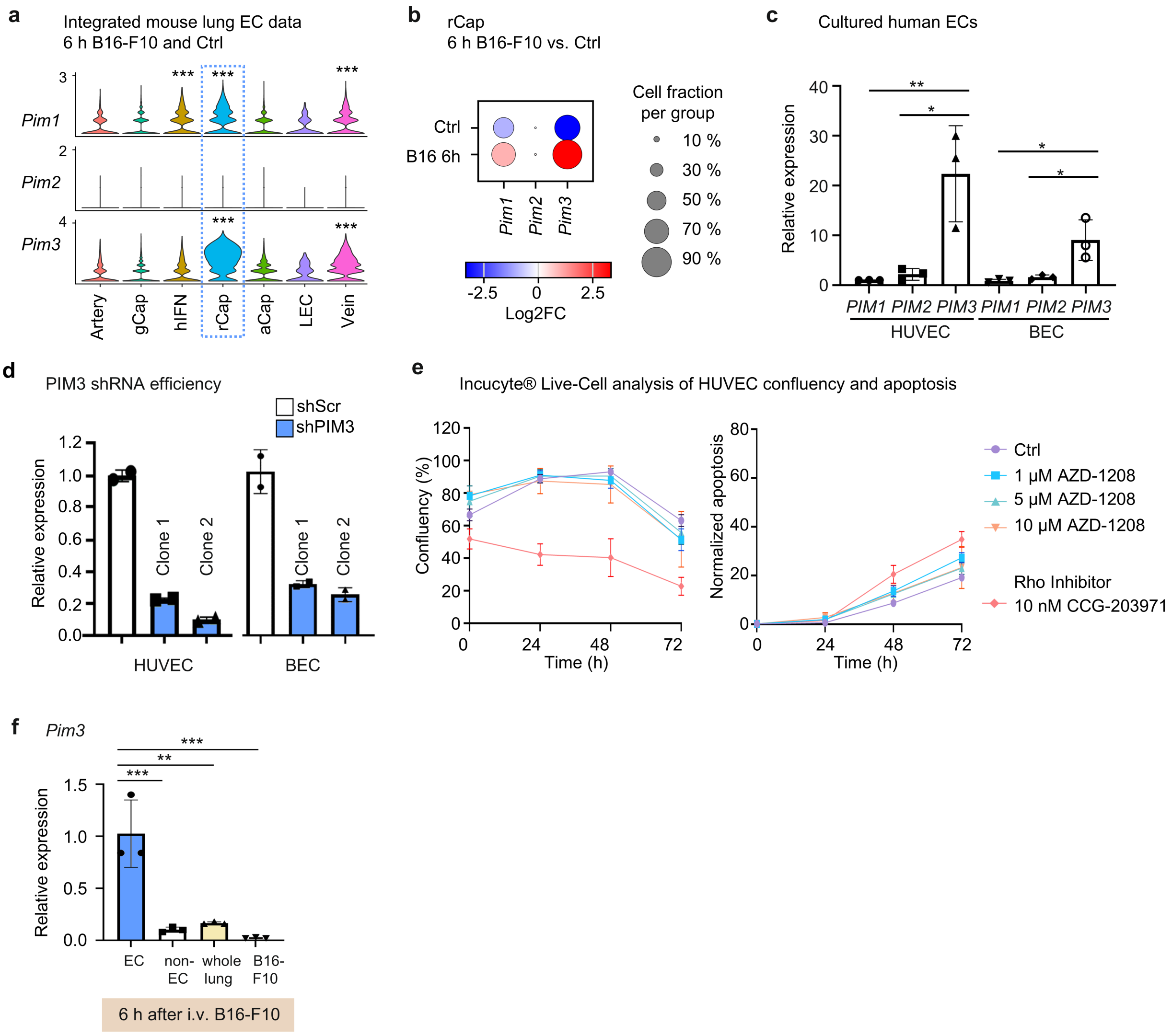

Supplementary Figure 10. PIM expression and effect of PIM inhibition in human ECs. **a**. *Pim* expression levels were visualized in the integrated scRNA-Seq data derived from mouse lungs 6 h after B16-F10 melanoma cell and PBS (Ctrl) intravenous (i.v.) injection. n= 3 independent experiments. **b.** Fold change of *Pim* expression in rCap 6 h after B16-F10 injection compared to control in scRNA-Seq data, $p < 0.01$ for *Pim1* and *Pim3*. **c**. Human umbilical vein ECs (HUVEC) and dermal microvascular blood ECs (BEC) were analysed using RT-qPCR for *PIM* expression. Values are normalized to *HPRT* and shown relative to *PIM1*. n=3 independent experiments. **d**. Efficiency of *PIM3* silencing after transfection of HUVECs using two different lentiviral vectors (shPIM3) by RT-qPCR as compared to scrambled control (shScr). Values are normalized to *HPRT*. **e**. HUVEC confluency and apoptosis were analysed using IncuCyte® in the presence of the PIM inhibitor AZD-1208 or 0.1% DMSO (Ctrl), as indicated, in reduced 2.5% serum. Rho GTPase inhibitor was used as a positive Ctrl. Apoptosis was calculated based on Caspase Green Dye counts normalized to confluency, performed in triplicate for all AZD-1208 and CCG-203971 concentrations. **f**. *Pim3* RT-qPCR (normalized to *Hprt*) from PECAM1+ EC and PECAM1- non-EC populations and GFP+ B16-F10 cells isolated from mouse lungs or total lung lysate, following injection of B16-F10 (500,000) i.v. n=3 mice per group. Wilcoxon rank-sum test to compare average expression (a, b), one-way Anova with multiple comparison, $p < 0.05^*$, $< 0.01^{**}$, $< 0.001^{***}$ (c, f). Data are presented as mean values +/- SD (c, f) or +/- SEM (e). Source data are provided as a Source Data file. *This is supportive data for Figures 5-7.*

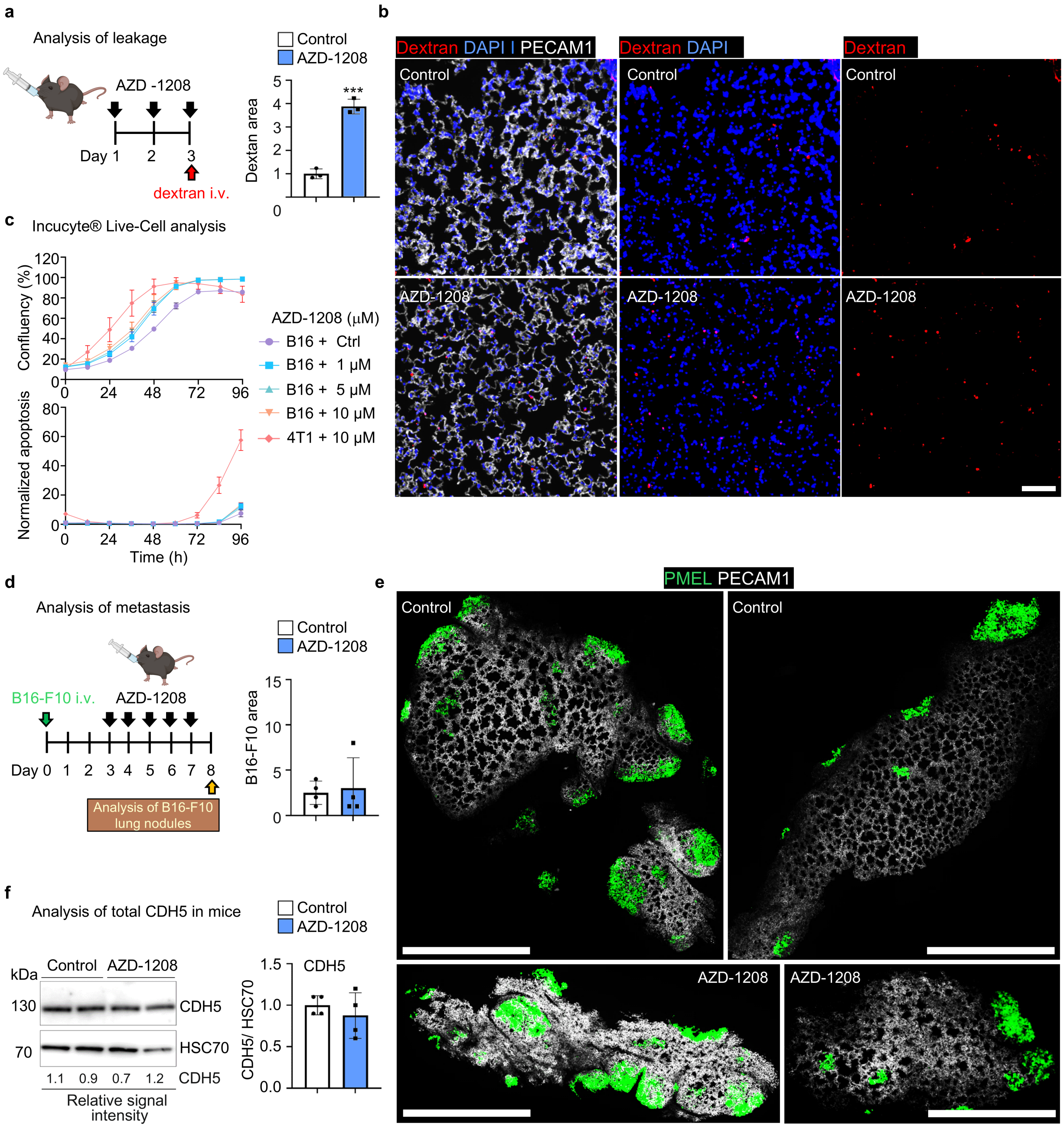

Supplementary Figure 11. PIM inhibition increases vascular leakage but does not affect melanoma cell growth. a. C57BL/6 mice were administered orally daily with AZD-1208 30 mg kg^{-1} or vehicle (Control), for three consecutive days. Leakage was measured by injecting 70 kDa fluorescent dextran intravenously (i.v.) for the last 10 minutes. Relative leakage normalized to tissue area was quantified from epifluorescent microscopy images of lung sections. n = 3 mice per group. **b**. Representative images of lung sections shown in a. **c**. Cultured B16-F10 and 4T1 melanoma cells were treated with AZD-1208 or 0.1% DMSO (Control), and imaged using IncuCyte® for cell confluency and apoptosis (Caspase Green Dye counts normalized to confluency). A representative experiment, performed in triplicate and independently repeated three times (n=3), is shown. Complete data of the 4T1 experiment is provided in Supplementary Fig. 12b. **d**. B16-F10 cells or PBS (Control) were injected i.v. into C57BL/6 mice. AZD-1208 was administered from day 3 onward until day 8, when the experiment was terminated. Melanoma cells were stained in lung sections for PMEL, and metastatic area per lung area was quantified. n=4 mice per group. **e.** Representative images from d. **f**. CDH5 signal was quantified from lung lysates from the experiment in d-e using immunoblotting, normalized to HSC70. n=4 mice per group. Two-tailed unpaired t-test (a, d, f), $p < 0.001$***. Data are presented as mean values +/- SD (a, d, f) or +/-SEM. (c). Scale bars 100 μm (b), 500 μm (e). Illustration created in Biorender.com (a, d). Source data are provided as a Source Data file. *This is supportive data for Figures 6-7.*

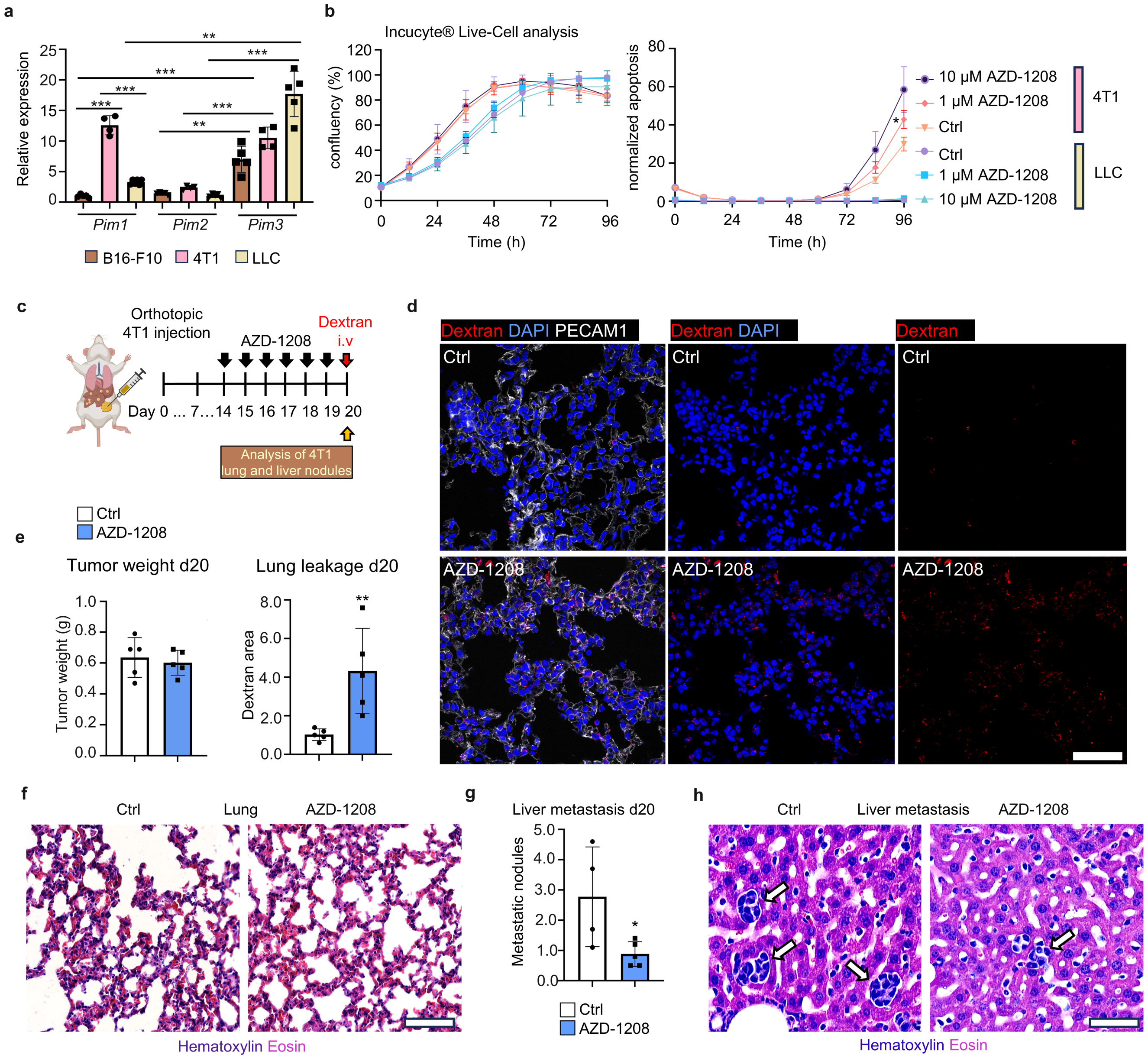

Supplementary Figure 12. PIM inhibition increases leakage in the lungs of 4T1 tumor bearing female Balb/c mice. a. RT-qPCR of cultured B16-F10 (n=5 independent experiment), 4T1 mouse mammary carcinoma (n=4) and Lewis lung carcinoma (LLC, n=5) cell lines. Values were normalized to *Hprt* and shown are results relative to *Pim1* expression in B16-F10. **b.** Cultured 4T1 and LLC cells were treated with AZD-1208 or 0.1% DMSO (Ctrl), and confluency and apoptosis were measured using IncuCyte® (Caspase Green Dye counts normalized to confluency). A representative experiment, performed in triplicate and independently repeated three times (n=3), is shown. **c.** 4T1 cells were orthotopically injected into the 4th mammary fat bad of Balb/c female mice. AZD-1208 or vehicle (Ctrl) was administered daily during 14-19 d post implantatation. 70 kDa fluorescent dextran was injected i.v. prior to sacrifice for 10 min for analysis of leakage. **d.** Representative images of dextran leakage in lung sections stained for PECAM1 and DAPI (nuclei), imaged using 3D Histech slide scanner. **e.** Tumor weight and relative leakage quantified from lung sections at the endpoint from experiment in d. n=5 mice per group. **f.** Representative images of Hematoxylin & Eosin (HE) stained lung sections. n=5 mice per group. **g.** Analysis of liver metastasis (nodules with > 5 cells were counted). n=4 mice for Ctrl, n=5 mice for AZD-1208 group **h.** Representative images of liver metastases in g, detected based on HE staining, indicated by arrows. One-way Anova (a) and two-way Anova (b) both with multiple comparison, two-tailed unpaired t-test (e, g) $p < 0.05$*, < 0.001**. Data are presented as mean values +/- SD (a, e, g) or +/- SEM. (b). Scale bar 50 μm (d, f, h). Illustration created in Biorender.com (c). Source data are provided as a Source Data file. *This is supportive data for Figure 7.*

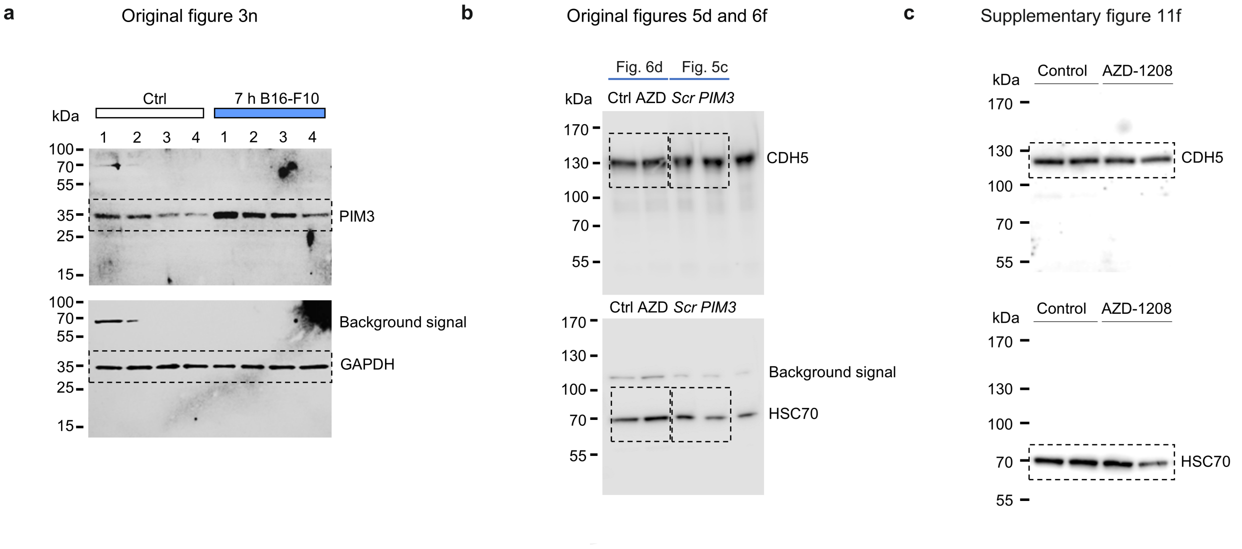

Supplementary Figure 13. Complete Western blots for figures 3n (a), 5d and 6f (b) and Supplementary figure 11f (c). **a.** Endothelial cells were isolated from lungs 7 h after B16-F10 or PBS injection (i.v.), and lysed. Lysates were detected using antibodies against PIM3 (Cell Signaling Technology #4165) and GAPDH (Cell Signaling Technology #97166). **b**. HUVEC treated with shPIM3 or shScr shRNA, or AZD-1208 or vehicle only (Ctrl) were lysed and detected using antibodies against CDH5 (BD Biosciences 555289) and HSC70 (Santa Cruz Biotechnology SC-7298). **c.** Mice were administered AZD-1208 or vehicle (Control), and lung lysates detected using antibodies against CDH5 (BD Biosciences 555289) and HSC70 (Santa Cruz Biotechnology SC-7298). Source data are provided as a Source Data file.

Santio et al. Endothelial Pim3 kinase protects the vascular barrier during lung metastasis.
Supplementary Information; Supplementary Tables 1-4

Supplementary Tables 1-4

Supplementary Table 1. Changes in the Jak-Stat pathway genes in metastatic vs control lung ECs. Changes in the Jak-Stat pathway genes in the metastatic lung ECs 6 h after i.v. injection of melanoma cells in mice as compared to lung ECs from control-treated mice. scRNASeq analysis of three independent datasets 1, 2 and 3. Shown are adjusted *p*-values (two-sided Wilcoxon Rank-Sum Test, significant p <0.05, non-significant ns).

**Gene**	**Artery**	**gCap**	**hIFN**	**rCap**	**aCap**	**LEC**	**Vein**
Jak1	*ns*	2,02E-49	0,00048	9,70E-10	1,07E-09	*ns*	0,02031
Jak2	*ns*	*ns*	*ns*	9,79E-21	*ns*	*ns*	*ns*
Jak3	*ns*	2,09E-20	*ns*	*ns*	*ns*	*ns*	0,02733
Stat1	*ns*	1,21E-43	0,19066	0,0115	1,11E-09	*ns*	*ns*
Stat2	*ns*	4,67E-21	*ns*	*ns*	*ns*	*ns*	*ns*
Stat3	*ns*	3,01E-83	1,19E-11	0,00018	1,51E-11	*ns*	*ns*
Stat4	*ns*	*ns*	*ns*	*ns*	*ns*	*ns*	*ns*
Stat5a	*ns*	*ns*	*ns*	*ns*	*ns*	*ns*	*ns*
Stat5b	*ns*	*ns*	*ns*	*ns*	*ns*	*ns*	*ns*
Stat6	*ns*	1,65E-05	*ns*	*ns*	*ns*	*ns*	*ns*

Supplementary Table 2. Primary antibodies.
Antibodies for immunocytochemistry (ICC),Western blotting (WB), immunohistochemistry (IHC), immunprecipitation (IP) and fluorescence-activated cell sorting (FACS), mouse (m) or human (h).

**Target**	**Manufacturer**	**Product code**	**Dilution**	**Staining**
m aSMA	Sigma-Aldrich	C6198	**1:350**	IHC
Actin (AlexaFluorTM 488 Phalloidin)	Thermo Fisher Scientific	A12379	1:40	ICC
h CTNNA (a.k.a. α-catenin)	Nagafuchi, A. & Tsukita, S. The Loss of the Expression of α Catenin, the 102 kD Cadherin Associated Protein, in Central Nervous Tissues during Development. *Dev. Growth Differ.* **36**, 59–71 (1994).[2]	-	1:300	ICC
h CTNNB (a.k.a. β-catenin), clone D10A8	Cell Signaling Technology	#8480	1:300	ICC
m COL4 (a.k.a. Collagen IV)	Bio-rad	2150-1470	1:400	IHC
m CD68	Bio-rad	MCA1957	1:400	IHC
h CTNND (a.k.a. δ-catenin), clone D752M	Cell Signaling Technology	59854	1:300	ICC
m GAPDH	Cell Signaling Technology	#97166	1:1500	WB
m PECAM1	Millipore	MAB1398	1:400	IHC, IP
m PECAM1 (a.k.a. CD31), PE-conjugated	BD Pharmingen	553373	1:300	FACS
m PIM3	Cell Signaling Technology	#4165	1:1000	WB
m PMEL	Abcam	Ab137078	1:300	IHC
m Podocalyxin	R&D	#AF1556	1:350	IHC
m PTPRC (a.k.a. CD45), APC-conjugated	BD Biosciences	561018	1:300	FACS
m S100A8	R&D	AF3059	1:300	IHC
m, h CDH5 (a.k.a. VE-cadherin)	BD Biosciences	555289	1:150	ICC, WB
m CDH5 (a.k.a. VE-cadherin)	eBioscience	14-1441	1:150	IHC
m VIM, clone V9	Santa Cruz Biotechnology	Sc-6260	1:200	IHC
m, h HSC70	Santa Cruz Biotechnology	SC-7298	1:10000	WB

Supplementary Table 3. Secondary antibodies.
Antibodies used for immunocytochemistry (ICC) and immunohistochemistry (IHC).

Antibody name	Manufacturer	Code	Dilution	Stainings
Donkey anti-mouse AlexaFluor® 594	Thermo Fisher Scientific	A21203	1:1000	IHC
Donkey anti-mouse AlexaFluor® 594	Thermo Fisher Scientific	A21203	1:300	ICC
Donkey anti-rabbit AlexaFluor® 488	Thermo Fisher Scientific	A21206	1:500-1000	IHC
Donkey anti-rabbit IgG AlexaFluor® 568	Thermo Fisher Scientific	A10042	1:1000	IHC
Donkey anti-rabbit IgG Alexa Fluor® 647	Thermo Fisher Scientific	A31573	1:500	IHC
Donkey anti-rat IgG AlexaFluor® 594	Thermo Fisher Scientific	A21209	1:300	IHC
Goat anti-armenian hamster Alexa Fluor® 647	Jackson Immunoreseach	127-605-160	1:300	IHC
Swine Anti-Rabbit Immunoglobulins/HRP	Dako	P0217	1:4000	WB
Goat anti-mouse HRP	Jackson ImmunoResearch	115-035-003	1:10000	WB

Supplementary Table 4. Primers for RT-qPCR.
Sequences of human and mouse primers used.

Target	Forward primer (5’>3’)	Reverse primer (5’>3’)
Adamts9	TCGTGTTCTGTCTCTTGTGGTCG	CTCTCTGGCTGCTTTGTGTGTC
Bcl3	AGGGACCTTTGATGCCCATTTA	CTGAGTATTCGGTAGACAGCGG
BCL3	CGGAGCCTTACTGCCTTTGTA	CCGTAGGTTGTTGTAGATGTCG
Inhbb	CAGGTGGAATGTGGTGGAGAA	CGATGAGCCGAAAGTCGATGA
INHBB	ACTCAACCTAGACGTGCAGTG	GGGTGCTATGATCCAGTCGTT
Mlana	TCCTGGGGATTGCTCTGCTT	CGGGCTGATGGGATTTCTCT
Osmr	ATGAGCGCAAACTACCTCTTCA	ATTCTCCGACCACACTTGTCTC
Pecam1	CTGCTGACCCTTCTGCTCTGTTC	GGCAGGCTCTTCATGTCAACACT
PIM1	TTCGGCTCGGTCTACTCAGG	GGCTCCCCTTTCCGTGATGA
PIM2	TGATTTTGGTTCTGGTGCCCT	TGTCATAGAGGAGGATGCCCA
Pim3	AAGCTCATCGACTTCGGCTC	CAGGGAGAGACACCACTCAATAAG
PIM3	AAAATCTGCTTGTGGACCTGC	GATCTCCTCGTCCTGCTCGAA
Pmel	TGGAAGACCTGGGGAAAATACTG	CTGAAGGGCAAAGATGAGAGGAT
Ptprc	CCTGAGCCTGTATCTAAACCTGA	CGGACGATCTGCTTTTGTGTG
Scarb1	TTTCTCAACACGTACCTCCCAG	GTTACACTGCTCTGAATGCCAA
Tmem252	TTGTGGCCTACGTGCTTCTG	AAGCTGGAGGGTAAAAGTCAGG

Supplementary Note 1

SYSTEM REQUIREMENTS

Windows Operating system

Computer with minimum:
-64 Gt RAM
-CPU with 8 cores or more
- 1 Tb of hard disk space

1: Install R (Version: 4.4.1)
2: Install RStudio (Version: 2024.04.2+764)

Install the following R packages:

BiocNeighbors	https://www.bioconductor.org/packages/release/bioc/html/BiocNeighbors.html
CellChat	http://www.cellchat.org/
ClusterProfiler	https://bioconductor.org/packages/release/bioc/html/clusterProfiler.html
ComplexHeatmap	https://jokergoo.github.io/ComplexHeatmap-reference/book/index.html
data.table	https://rdatatable.gitlab.io/data.table/
DoubletFinder	https://github.com/chris-mcginnis-ucsf/DoubletFinder
dplyr	https://dplyr.tidyverse.org/
enrichplot	https://bioconductor.org/packages/enrichplot.
ggalluvial	https://corybrunson.github.io/ggalluvial/
ggplot2	https://ggplot2.tidyverse.org/
glmGamPoi	https://github.com/const-ae/glmGamPoi
heatmaply	https://talgalili.github.io/heatmaply/
htmltools	https://rstudio.github.io/htmltools/
Igraph	https://r.igraph.org/
maftools	https://bioconductor.org/packages/devel/bioc/vignettes/maftools/inst/doc/maftools.html
magrittrhttps://magrittr.tidyverse.org/	
nichenetr	https://github.com/saeyslab/nichenetr
Matrix	https://rdrr.io/rforge/Matrix/
NMF	https://renozao.github.io/NMF/devel/index.html
patchwork	https://patchwork.data-imaginist.com/
pathview	https://pathview.r-forge.r-project.org/
plotly	https://plotly.com/r/getting-started/
RColorBrewer	https://r-graph-gallery.com/38-rcolorbrewers-palettes.html
Reticulate	https://rstudio.github.io/reticulate/
scCustomize	https://samuel-marsh.github.io/scCustomize/index.html
rstatix	https://rdrr.io/cran/rstatix/
Seurat	https://satijalab.org/seurat/
SeuratDisk	https://github.com/mojaveazure
tidyverse	https://www.tidyverse.org/
Ucell	https://rdrr.io/github/carmonalab/UCell/
viridis	https://cran.r-project.org/web/packages/viridis/vignettes/intro-to-viridis.html

INSTALLATION QUIDE

https://posit.co/download/rstudio-desktop/
estimated install time: 30 min - 1 h

See links above for R package installation.

INSTRUCTIONS FOR USE

DATA 1. Mouse melanoma and control lung data

Download data:
https://www.ncbi.nlm.nih.gov/geo/query/acc.cgi?acc=GSE235394
https://www.ncbi.nlm.nih.gov/geo/query/acc.cgi?acc=GSE253749
https://www.ncbi.nlm.nih.gov/geo/query/acc.cgi?acc=GSE253751

Follow code at
https://github.com/nmsantio/Mouse-melanoma-lung-EC-scRNASeq
Step 1. Mouse Melanoma Data reading

prior to beginning install all packages listed above and name the data folders accordingly:

main folder:
exp1

subfolders:
c (063barcodes.tsv.qz, 063features.tsv.gq, 063matrix.mtx.gz)
6h (064barcodes.tsv.qz, 064features.tsv.gq, 064matrix.mtx.gz)

main folder:
exp2

subfolders:
c (052barcodes.tsv.qz, 052features.tsv.gq, 052matrix.mtx.gz)
6h (051barcodes.tsv.qz, 051features.tsv.gq, 051matrix.mtx.gz)
28h (053barcodes.tsv.qz, 053features.tsv.gq, 053matrix.mtx.gz)

main folder:
exp3

subfolders:
c (058barcodes.tsv.qz, 058features.tsv.gq, 058matrix.mtx.gz)
6h (059barcodes.tsv.qz, 059features.tsv.gq, 059matrix.mtx.gz)

Step 2. Find Doublets
Step 3. Data integration and clustering

DATA 2. Mouse lung tumor EC data from
https://endotheliomics.shinyapps.io/lung_ectax/

Download data:
Mouse lung: click to select
Click to download the data, metadata and information

Follow code at
https://github.com/nmsantio/Create-Seurat-Objects-from-on-line-sources

Step 1. Lung Tumor EC Tax data to Seurat object
Step 2. Recluster LungECTax data

DATA 3. Human lung EC data from
https://hlca.ds.czbiohub.org/

Download data:
https://www.synapse.org/Synapse:syn21041850/wiki/600865

Follow code at:
https://github.com/nmsantio/HLCA_scRNASeq_EC
human EC clustering

4. Integrate human data from DATA 3. with mouse data from DATA 1.

Follow code at:
https://github.com/nmsantio/Integration-of-mouse-and-human-scRNASeq-data

Open both datasets created from DATA 3 and DATA 1 in R.
Step 1. Downsampling - Replace filename with mouse data file name
Step 2. Mouse gene names to human
Step 3. Data integration - Check that the human data file has name hLung, if not, replace hLung with the datafile name

Link summary to github
https://github.com/nmsantio/Mouse-melanoma-lung-EC-scRNASeq
https://github.com/nmsantio/Create-Seurat-Objects-from-on-line-sources
https://github.com/nmsantio/HLCA_scRNASeq_EC
https://github.com/nmsantio/Integration-of-mouse-and-human-scRNASeq-data

Additional visualization instructions can be found from the above mentioned links, mainly at
https://satijalab.org/seurat/
https://samuel-marsh.github.io/scCustomize/index.html

Santio et al. Endothelial Pim3 kinase protects the vascular barrier during lung metastasis.
Supplementary Information; Supplementary references

Supplementary References

1. Goveia, J. *et al.* An Integrated Gene Expression Landscape Profiling Approach to Identify Lung Tumor Endothelial Cell Heterogeneity and Angiogenic Candidates. *Cancer Cell* 37, 421 (2020).

2. Nagafuchi, A. & Tsukita, S. The Loss of the Expression of α Catenin, the 102 kD Cadherin Associated Protein, in Central Nervous Tissues during Development. Dev. Growth Differ. 36, 59–71 (1994).
